# Improvements and Persisting Challenges in COVID-19 Response Compared with 1918–19 Influenza Pandemic Response, New Zealand (Aotearoa)

**DOI:** 10.3201/eid2909.221265

**Published:** 2023-09

**Authors:** Jennifer Summers, Amanda Kvalsvig, Lucy Telfar Barnard, Julie Bennett, Matire Harwood, Nick Wilson, Michael G. Baker

**Affiliations:** University of Otago, Wellington, New Zealand (J. Summers, A. Kvalsvig, L. Telfar Barnard, J. Bennett, N. Wilson, M.G. Baker);; University of Auckland, Auckland, New Zealand (M. Harwood)

**Keywords:** epidemiology, pandemics, influenza, H1N1, COVID-19, SARS-CoV-2, severe acute respiratory infection coronavirus 2, zoonoses, viruses, respiratory infections, New Zealand, Aotearoa, pandemic planning, response

## Abstract

Exploring the results of the COVID-19 response in New Zealand (Aotearoa) is warranted so that insights can inform future pandemic planning. We compared the COVID-19 response in New Zealand to that for the more severe 1918–19 influenza pandemic. Both pandemics were caused by respiratory viruses, but the 1918–19 pandemic was short, intense, and yielded a higher mortality rate. The government and societal responses to COVID-19 were vastly superior; responses had a clear strategic direction and included a highly effective elimination strategy, border restrictions, minimal community spread for 20 months, successful vaccination rollout, and strong central government support. Both pandemics involved a whole-of-government response, community mobilization, and use of public health and social measures. Nevertheless, lessons from 1918–19 on the necessity of action to prevent inequities among different social groups were not fully learned, as demonstrated by the COVID-19 response and its ongoing unequal health outcomes in New Zealand.

The world is continuing to experience the COVID-19 pandemic, which has resulted in >767 million reported cases and ≈6.9 million deaths (≈870 deaths/1 million persons) through June 2023 (*1*). Those numbers are likely a huge undercount; mortality has been estimated to be >3 times higher (*2*). New Zealand (Aotearoa, the commonly used Indigenous Māori language name for the country) experienced ≈2.4 million confirmed COVID-19 cases and ≈3,077 COVID-19 attributed deaths (≈597 per million population) reported up to mid-June 2023 (*3*). The country has also experienced severe effects of the COVID-19 pandemic through disruptions to the healthcare system and economy and wider societal harms (*4**–**7*). However, in terms of deaths, the influenza pandemic of 1918–19 still remains “New Zealand’s worst recorded natural disaster” (*8*).

The 1918–19 influenza pandemic occurred in the final stages of World War I (WWI) and is estimated to have killed 50–100 million persons worldwide, equaling >1% of the world’s population (*9*). This particularly lethal strain of influenza A(H1N1) virus spread to almost all parts of the globe, leaving just a few isolated locations untouched. In New Zealand, the 1918–19 influenza pandemic spread the length of the country through railway and shipping routes and is estimated to have killed >9,000 persons (*8*). The effects of this pandemic were severe, stressing the existing healthcare system (already stretched by the war effort) and, as in other nations, affecting all aspects of daily life and compounding existing societal and economic inequities.

Past pandemics provide insight into how societies, governments, and communities are affected and how they might respond to an emerging disease threat. Indeed, failure to examine past pandemic experiences limits our understanding and reduces the clarity of evidence and justification for future pandemic management and control. Given this background, we completed a historical review (Appendix) to consider how this island nation responded to these 2 severe pandemics and to explore whether ongoing lessons exist that are relevant both for today and for future pandemic planning.

## 1918–19 Influenza Pandemic in New Zealand

**Figure 1:**
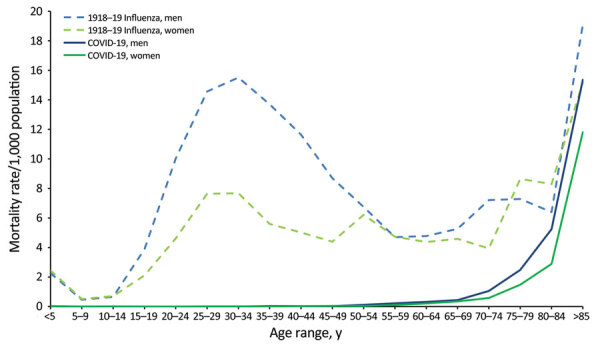
Cumulative mortality rate (deaths/1,000 population) in New Zealand (Aotearoa) during the 1918–19 influenza pandemic (for European-origin persons) and during the COVID-19 pandemic (all origins), by age and sex. The 1918–19 pandemic mortality data cover the entire period of the pandemic in NZ and are reproduced from Summers (*10*) and derived/approximated from publicly available sources (*8*; https://www3.stats.govt.nz/New_Zealand_Official_Yearbooks/1924/NZOYB_1924.html). Mortality data from 1918–19 for the Māori population are not available; therefore, mortality rates are likely underestimates. COVID-19 mortality data cover the period of January 2020–December 31, 2022. Mortality data were provided by the New Zealand Ministry of Health/Manatū Hauora, and population totals were sourced from Stats NZ/Tatauranga Aotearoa (https://www.stats.govt.nz/topics/population). Death was classified as a COVID-19 death when COVID-19 was the underlying cause of death or a contributory cause of death. The figure does not include 3 deaths with missing demographic information or the 589 deaths that were unclassified as of December 31, 2022 (and might subsequently be classified as COVID-19 deaths).

The first, relatively mild, wave of the 1918–19 influenza pandemic spread in New Zealand during July–October 1918. The more virulent second wave largely occurred during November–December 1918 (Appendix Table 1, Figure 1, panel A). Most pandemic deaths in New Zealand occurred during this second wave, which spread nationwide in a matter of weeks; some localized examples of prevention measures, such as quarantine and travel restrictions, have been documented (*8*). Vaccine use for bacterial pathogens during this pandemic is documented in New Zealand and in overseas-based New Zealand military personnel, who were part of vaccine studies (*11*). Some limited international evidence of vaccine efficacy for influenza-associated bacterial pneumonia (a common secondary infection) during this pandemic exists, but there was no coordinated distribution of vaccines to the public in New Zealand. This pandemic had a profound effect on children in New Zealand, not only as a result of influenza infection itself but also through detrimental effects on family and caregiving structure and by deaths of caregivers that left children orphaned (*8*). Evidence also exists for a sudden decrease in the annual birth rate in the country in 1918 and particularly 1919, a possible result of the association between influenza infections, social effects, and stillbirths or fetal loss (*12**,**13*).

In late 2018, we published a systematic review of all known literature on the experience of the 1918–19 influenza pandemic in New Zealand (*12*). We found epidemiologic patterns among residents during this pandemic that were consistent with international literature, such as a w-shaped age distribution for deaths (Figure 1) (*8**,**14**,**15*). Mortality rates were high among Indigenous Māori civilian and military populations compared with the European-origin population (*8**,**16*), and risk for death was higher among New Zealand military personnel who had a preexisting chronic disease or were recent military recruits (*8**,**15**–**17*). Unique findings focused on the novel risk factors for death, such as larger chest size in men (possibly an indicator of a different immune system response in men with larger bodies) (*17*) and lack of difference between mortality rates in men and women in the Māori population. The lack of difference in mortality rates by sex contrasted with the relatively higher death rates of men than women in the European-origin population in New Zealand (as was found in many other countries) (*12**,**15**,**18*). Although this H1N1 influenza virus was considered endemic by 1920, it continued to cause more severe influenza seasons for several more years, and long-term sequelae from the pandemic strain have been documented internationally (*19**,**20*) (Appendix).

## COVID-19 in New Zealand

The first identified case of COVID-19 in New Zealand was reported on February 28, 2020; the first outbreak peaked in March 2020 alongside the first national stay-at-home order (lockdown), border closures for noncitizens, and introduction of wide-ranging public health protections (Appendix Figure 1, panel B). The government initially adopted an elimination response strategy to manage the pandemic, which required tight border management to prevent the importation of COVID-19 cases and systems to extinguish outbreaks if they occurred (*21*).

Relatively small COVID-19 outbreaks occurred in 2020 and 2021 because of incursions coupled with new COVID-19 variants (*3**,**22*). In response, local (including iwi [tribal]–led), regional, and national public health and social measures (including lockdowns) were put in place to contain community spread. During those periods, businesses were closed, work was restricted unless deemed essential, and the government provided some financial assistance to businesses and employees.

A switch from an elimination strategy to a suppression strategy occurred in late 2021 during the Delta variant wave with the introduction of the COVID-19 Protection Framework (*21**,**23*). This framework focused on vaccination requirements for various indoor and public venues and included some limited travel restrictions. However, the framework was retired mid-September 2022, and only limited public health protections, such as mask-wearing in healthcare facilities, remained in place. The pandemic plan in New Zealand at the emergence of COVID-19 was (and remains as of mid-June 2023) based on a hypothetical influenza pandemic and predominantly uses a mitigation strategy (*24*). Therefore, the applicability of this plan to the characteristics of COVID-19 has been questioned (*4*).

Compared with other high-income countries, New Zealand experienced decreased excess winter deaths, a net decline in overall deaths, and an increase in life expectancy during the first 2 years of the COVID-19 pandemic (*25*). The largest waves to date in terms of cases, hospitalizations, and deaths have been from the Omicron variant (and its sublineages), which began in early 2022 and spread nationwide (*26*). By mid-June 2023, a total of 3,077 estimated deaths attributed to COVID-19 had occurred in the country (*3*).

The effects of COVID-19 in New Zealand have varied; the burden of hospitalizations and deaths have disproportionately affected Māori and Pacific persons (another ethnic grouping), and those groups have had lower rates of COVID-19 vaccination (although the difference varies by age group) (*3**,**6*). As of June 9, 2023, ≈89.3% of the total eligible New Zealand population had received 2 vaccine doses, and ≈73.2% had received >1 booster (third) vaccine dose (*3*). The pandemic has also had a major effect on children and adolescents because of widespread disruption to education at all ages (*27*).

Just over a year into the COVID-19 pandemic, the New Zealand government confirmed that the health system would be restructured to create 1 national service delivery organization to function alongside the continuing Ministry of Health (focused on policy), a dedicated Public Health Agency, and a Māori Health Authority (https://www.futureofhealth.govt.nz). The transformed health system aims to create a “more equitable, accessible, cohesive and people-centered system that will improve the health and wellbeing of all New Zealanders” (https://www.futureofhealth.govt.nz). This health system restructure was planned before the COVID-19 pandemic, however; unlike the health system restructuring and legislative changes that occurred in New Zealand after the 1918–19 influenza pandemic, this restructuring began during the COVID-19 pandemic.

## Comparison of 2 Pandemics

We identified key similarities and differences between hazards and responses across the 2 pandemics (Table). Both pandemics occurred among largely immunologically naive populations (with some exceptions in 1918–19) (*43*), and large proportions of the population were infected with marked ethnic health disparities, manifesting as higher rates of illness, hospitalization, and death, among Māori and Pacific peoples.

**Table:** Comparative summary of distinct features of 1918–19 influenza pandemic and the COVID-19 pandemic hazard and responses, New Zealand*

1918–19 influenza pandemic	COVID-19 pandemic	Similarities
Hazard and effects (both globally and in NZ, where data available)		
Caused by influenza virus H1N1	Caused by SARS-CoV-2	Likely zoonotic origins for the pandemic viruses
RNA virus that showed relatively slow genetic drift through mutation	Global infection fatality risk of 0.1%–2.0% up to June 2021 (*28*); NZ infection fatality risk 0.79% (estimated, January 2021 before vaccination) (*29*)	Transmitted between humans as a respiratory viral pathogen
Probably originated in domestic and wild birds (*30**,**31*)	RNA virus showing rapid genetic shifts through mutation and recombination, including within-host evolution during chronic infection of immunocompromised patients (*32*)	Immunologically naive population
Moderately transmissible, with R_0_ estimated at 2.4–4.3 (*33*)	Probably originated in bats (*31*)	High proportion of population infected
Incubation period of ≈a few hours to 2 d reported in a large US civilian hospital in 1918 (*34*) and general influenza estimates of 1–4 d (*35*)	Highly transmissible with estimated R_0_ of 9.5 for Omicron variant (*36*)	Marked ethnic health disparities experienced globally. For example, in NZ, notably higher death rates in the Māori population
Global case-fatality risk ≈1–2.5% (*20**,**37*)	Incubation period estimates differ by variant, with one meta-analysis reporting a pooled mean incubation time of 6.6 d (*38*)	Higher death rates in men internationally
Global infection fatality risk >2% (*28*)	Global estimate for case fatality risk of 1.12% as of July 26, 2022 (*1*). NZ case-fatality risk of 1.15 in 2020 (before vaccines), reduced to 0.09% as of July 2022 (with high vaccine coverage) (*3*)	Post-acute infection syndrome common
Infection gives long-term immunity (*39*)	Infection gives protection that fades over ≈3 y (*40*)	
Net effect is symptomatic infection in ≈8% of population each year (*41*)	Net effect is reinfections are common (*3*)	
Short, intense pandemic wave, with some smaller waves in subsequent years	Repeated, prolonged pandemic waves	
Relatively more severe illness in young adults and elderly	Relatively more severe illness in elderly and immunosuppressed	
Devastating spread of infection from NZ to surrounding Pacific nations	Regional border quarantine measures probably limited spread from NZ to South Pacific jurisdictions	
Response in NZ		
Lack of strategic response	Highly strategic national control response (elimination for first 20 mo of pandemic) with vigorous public communication	Large community/voluntary sector mobilization
No use of external border controls	Use of tight external border controls (in the first 2 years)	Use of physical distancing through closure of public facilities, businesses, schools, and cancellation of large public events, although less systematically in 1918–19
No specific test for pathogen available	Accurate diagnostic test and organized testing program	Some use of internal border controls
Limited use of case isolation and contact quarantine	Active contact tracing and quarantining of contacts	No specific curative treatment initially (although supportive management and treatment options for COVID-19 sufferers were developed, including antivirals)
Limited infection control in institutions	Infection prevention and control in health care and aged care	Iwi, hapū and marae-led care and support† (*7**,**8**,**42*)
No specific vaccine available	Highly effective vaccines in late 2020 (within 1 year)	Royal Commissions of Inquiries to investigate pandemic responses
Lack of economic and social support from government	Extensive economic and social support from government	
No widespread mask-wearing	Requirements (mandates) to use masks in some settings to limit transmission	

*For greater detail of the hazards, response, and various impacts of the two pandemics in NZ, see Appendix Table 1. NZ, New Zealand; R_0_, basic reproductive number. †Indigenous Māori language terms: iwi refers to tribe and hapū refers to subtribe. Marae (meeting grounds) are the focal point of Māori communities and are a complex of carved buildings and grounds that belongs to a particular iwi, hapū, or whānau (family).

Both viruses are moderately to highly infectious; basic reproductive numbers (R_0_) were estimated to be >2.4 (Table) (*37**,**42*). A key difference is that the incubation period (and serial interval) is much shorter for influenza. An estimate of the incubation period for 1918–19 influenza is a few hours to 2 days (*34*); for influenza A, 1.4 days (*35*). For SARS-CoV-2, by contrast, one mean estimate of incubation is 6.57 days (*38*). The longer incubation period for COVID-19 has made contact tracing and quarantine of contacts much more feasible.

The 1918–19 influenza pandemic caused a short, intense pandemic wave with high death rates that swept through New Zealand in <2 months (November–December 1918) and likely infected ≈50% of the population (*8*). The first Omicron variant wave of the COVID-19 pandemic moved through New Zealand in a similarly short period (February–April 2022). Unlike the 1918-19 influenza pandemic, it was followed by a succession of waves; a second occurred in June–August 2022, a third began in November 2022, and a fourth began in April 2023. These waves were each dominated by different Omicron subvariants (BA.1 and BA.2 for the first wave, BA.4 and BA.5 for the second, and a mix of multiple Omicron subvariants in the third and fourth waves) (*3*). Influenza H1N1 (such as the 1918–19 influenza virus) and SARS-CoV-2 are RNA viruses that mutate more readily than DNA viruses (*44*). However, SARS-CoV-2 has demonstrated a capacity for sudden and frequent antigenic shifts that result in new variants and subvariants with multiple mutations, which enables it to escape existing immunity and cause high levels of reinfection and a succession of pandemic waves (*32*). One change in human populations between 1918–19 and 2020 onward is the likely increase in the proportion of persons now living with known immune suppression. SARS-CoV-2 appears able to cause chronic infections in such patients, during which it can have rapid within-host evolution (*32*).

Of note, the lethality of H1N1 in 1918–19 (global infection fatality risk >2%) overlapped with the range reported for SARS-CoV-2 (global infection fatality risk 0.1%– 2%) before vaccines were introduced (*28**,**29*). After widespread COVID-19 vaccination, the case-fatality risk in New Zealand dropped by an order of magnitude, from 1.15% in 2020 to ≈0.13% by the end of May 2023 (*3*). This decline might also reflect the reduced severity of the Omicron variant relative to the Delta variant, although Omicron appears to have similar virulence to the original variant that dominated during the first year of the COVID-19 pandemic (*45*). Furthermore, immunity after infection with H1N1 virus in 1918–19 appeared to be long-lasting (*39*). By contrast, immunity against infection generated by SARS-CoV-2 appears to fade over ≈3 years (*40*). In addition, this immunity is much less effective at preventing infection with subsequent COVID-19 subvariants, although protection against severe infection appears to be well sustained after both natural infection and vaccination (*40*).

We observed a w-shaped distribution of deaths in New Zealand during the 1918–19 pandemic that was more pronounced for men than women in almost all age groups (Figure 1). However, we observed no evidence of a w-shaped distribution of deaths by age for COVID-19 in New Zealand; the mortality rate increased exponentially with older age. The rate of overall attributable deaths was higher among men than women, which is consistent with international findings (*3**,**46*). For both pandemics, higher mortality rates were observed in specific populations, such as Māori and Pacific peoples (*3**,**6**,**8*). Reported rates of COVID-19 illness have been generally higher among children and younger adults in New Zealand (*3*). However, this difference might reflect increased exposure to infection because they have higher levels of social contact than older adults; rates of self-reporting among the younger population could also be higher.

A wide-ranging government response with robust community mobilization was observed during both pandemics, as was a substantial reliance on charitable contributions to support persons and communities (Appendix Table 1) (*4**,**8**,**47*). Physical distancing measures and travel/border restrictions were used in both pandemics, but public health protections were far tighter during the COVID-19 pandemic (particularly during 2020 and 2021). Additional external border controls used the advantage of New Zealand being a remote island nation and having a brief window of time to implement controls before widespread domestic COVID-19 transmission occurred. However, during 1918–19, use of internal border restrictions was limited and inconsistent, and no substantial external travel restrictions or border control was in place. For example, a discriminatory travel ban on public transport for Māori (unless issued a health permit) was implemented, and other unofficial bans were extended to other premises, such as business places (*8*).

Institutional infection control and prevention was limited during 1918–19, although some temporary hospitals were established for influenza patients, in addition to separate hospitals for Māori patients (*8*). The response in 1918–19 was unlike the response during COVID-19, in which extensive prevention and control measures were used in a range of healthcare and aged-care settings and integrated into the initial Alert Level System and the subsequent COVID-19 Protection Framework (*21**,**47*).

## Discussion

More than a century has now passed since the 1918–19 influenza pandemic, but it remains the worst public health disaster in recorded New Zealand history. More than 9,000 influenza deaths occurred in just a couple of months, and during the final stages of WWI, New Zealand residents faced a uniquely difficult period in the nation’s history. In particular, the Māori population was disproportionally affected by the pandemic, and many Māori pandemic deaths probably remain undocumented (*8*). The response during and after this period provides insight into how New Zealand society might respond to future disease threats, as well as to the continuing COVID-19 pandemic.

Probably the most fundamental difference in responses to COVID-19 and influenza was the use of a national control strategy, namely an elimination strategy for SARS-CoV-2 (*48*). The early use of the elimination strategy in New Zealand in 2020 helped maintain a relatively low death rate in the first 2 years and reduced the economic impact of the COVID-19 pandemic compared with other nations (*1*). New Zealand also observed an increase in life expectancy during this period (*25*) and low estimates of excess deaths compared with a pre–COVID-19 period (≈0.02% as of May 2023), unlike other high-income nations, such as the United States (12.8%), United Kingdom (10.0%), and Sweden (5.1%) (*1*). This proactive response to COVID-19 is markedly different from 1918–19, when no clear strategy was implemented for preventing or managing the influenza pandemic, resulting in substantial deaths and reduced birth rates in the following years (*12**,**13*).

The death patterns observed in 1918–19 highlighted health inequities and the factors driving them, such as household crowding, comorbidities, and unequal access to healthcare. Reasons for poorer health outcomes among Māori are complex; Māori persons in 1918–19 experienced higher rates of chronic disease (compared to the European-origin population in New Zealand), barriers in access to healthcare, and discriminatory outbreak management approaches. For example, in 1918–19, the Māori population had a substantially higher pandemic influenza mortality rate of 42.3 per 1,000 compared with 5.8 per 1,000 among the European-origin population; as a result, in the final 2 months of 1918, an estimated 4% of the Māori population died from pandemic influenza (*8*).

Those health inequities persist today (*16*). Although the New Zealand government has acknowledged failings in the COVID-19 pandemic response and provided some targeted support to Māori providers (and other services such as those for Pacific and disabled persons), cases, hospitalizations, and death rates for COVID-19 have been disproportionally higher among those groups (*3*). Rates of COVID-19 vaccination are also lower among Māori adults and children than among other ethnic groups. Therefore, the principles of equity, partnership, and active protection, as guaranteed in the Te Tiriti o Waitangi–Treaty of Waitangi between the Government (Crown) and Māori, continue to be inadequately addressed 100 years after the first pandemic. Fortunately, some of this deficit was addressed through Māori-led initiatives during the COVID-19 pandemic, such as basic living support (for example, food parcels to families [*7*]) and health service provision (for example, testing and vaccination drives by community groups, with or without government support). Several iwi (tribes) also initiated border controls for their tribal areas, emulating the approaches used in 1918–19 to limit the spread and severity of disease and thus protect their whānau (families) and communities.

When comparing the 2 pandemics, considering how scientific understanding has progressed and given us better ways of identifying, measuring, and describing the effect of infectious diseases is key. For example, the first human influenza virus was not isolated until 1933, more than a decade after the 1918–19 influenza pandemic (*8*). One distinct research area is the growing awareness of post–acute illness effects. The long-term effects of COVID-19 infection, which include both post–acute infection syndrome (long COVID) and organ system–specific effects (manifesting as excess deaths for at least 1 year after acute infection), appear to be relatively common. Long-term effects after the 1918–19 influenza pandemic were recognized, but fewer scientific tools to investigate them existed (*19*). Recent comparisons of COVID-19 with influenza suggest that sequelae from influenza appear less common (*49*).

**Figure 2:**
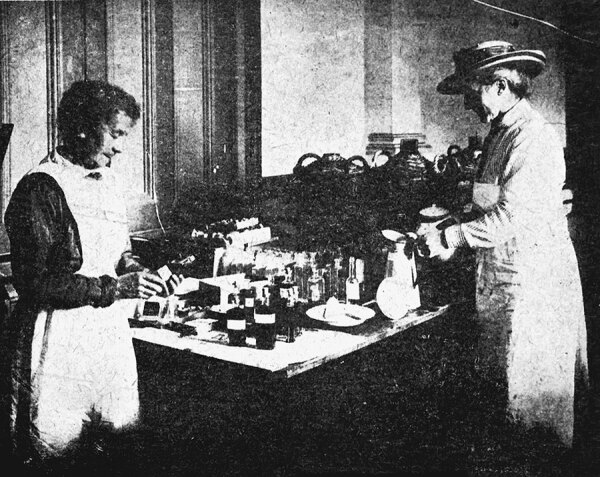
Medicine department at the Wellington Town Hall during the 1918 influenza epidemic. Shows where the standard mixture and tonic were prepared and bottled. Mrs. Waters (right) was in charge. Taken by an unidentified photographer. Reproduced from New Zealand Free Lance: 1/2-C-016207-F, 1918, Alexander Turnbull Library: National Library of New Zealand, Wellington, New Zealand.

**Figure 3:**
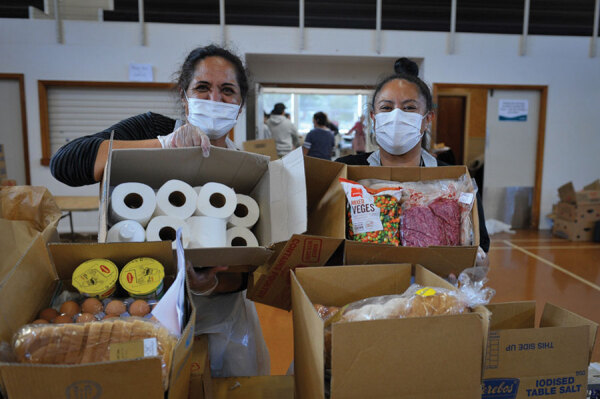
Workers at Kōkiri Marae preparing food and sanitation packages for the Lower Hutt and Wainuiomata communities during COVID-19 pandemic, New Zealand. Photograph by Luke Pilkinton-Ching, University of Otago, Wellington, New Zealand.

During 1918, WWI was continuing to have a substantial impact on daily life; ≈40% of the New Zealand adult male population served in the military during the war, and many doctors and nurses were stationed overseas. This huge disturbance to normal life meant that when the pandemic hit, fewer able-bodied adults were available in traditional roles to provide assistance, and this gap was compounded by the higher rates of illness and death in younger adults. Therefore, many other residents stepped up to help by volunteering in temporary hospitals, providing food and medical supplies, transporting those who were ill, and serving on block committees that managed and supported local communities by coordinating relief (Figure 2) (*8*). Numerous examples of children playing essential roles during the 1918–19 pandemic by delivering supplies and working in hospitals have also been detailed (*8*). Similar examples were observed during the COVID-19 pandemic; local communities provided food and other supplies throughout New Zealand (Figure 3) (*7*), and children in secondary schools took employment in essential roles in supermarkets while schools were closed to support their families and fill labor shortages. The government also provided economic assistance during COVID-19, although this assistance was intermittent and was particularly focused on localities experiencing the tightest controls.

Unlike the 1918–19 influenza pandemic, which was largely over in 2 months, the COVID-19 pandemic has sustained itself globally for >3 years. Consequently, the effect of the COVID-19 pandemic on societal cohesion in New Zealand might be different from that observed during 1918–19; the ongoing COVID-19 response, vaccine provision and mandates, and overall management by the government has led to increased displays of social division. This division suggests the ongoing need for a more equitable and effective pandemic response, at both national and international levels.

Surprisingly, after 3 years of the COVID-19 pandemic, New Zealand still lacks a generic pandemic plan, and little evidence of planning for future disease threats (other than COVID-19 or influenza) exists (*47*). Therefore, it appears that New Zealand has not yet fully learned the lessons of 1918–19; the COVID-19 response has largely taken a reactive approach to new challenges, rather than a proactive stance (*47*). A more proactive approach could have implications for controlling other infectious diseases (for example, improving infrastructure to support improved public health and social measures) and managing COVID-19 aftereffects such as long COVID and long-term effects on children.

Restructuring the health system during the COVID-19 pandemic might not have been optimal timing and is unlikely to incorporate all potentially relevant lessons from the entire period of the pandemic, unlike the restructuring after 1918–19. A Royal Commission of Inquiry investigating the response in New Zealand to the COVID-19 pandemic was announced in December 2022, but the scope of the inquiry is constrained. It excludes, for example, any assessment of the effect of the health system reforms, the epidemiology of the COVID-19 virus, private sector involvement, or various judgments and decisions related to the pandemic in various courts and independent agencies. A major positive feature is its focus on improving future pandemic preparedness (*50*).

New Zealand’s “team of 5 million,” as former Prime Minister Jacinda Ardern voiced in 2020 in reference to the population, is arguably now somewhat fractured by the prolonged COVID-19 pandemic and spread of the Omicron variant. Every aspect of the pandemic response has also been scaled back, with less use of public health and social measures and slowing uptake of vaccination and boosters. Therefore, it is difficult to identify, from a public health perspective, the government’s ongoing strategy for managing COVID-19, how persisting inequities associated with infection are to be addressed, or how those most at-risk are to be protected. However, it is worth remembering that New Zealand emerged from the devastating 1918–19 influenza pandemic by strengthening its health system with the goal of learning lessons from its pandemic response. At this point, there remains an opportunity for New Zealand, and the rest of the world, to build capacity to prevent future pandemics and to better respond to them when they are unavoidable.

Appendix Additional information about improvements and persisting challenges in COVID-19 response compared to 1918 influenza pandemic response, New Zealand (Aotearoa)
