# Supplementary material for: Improvements and Persisting Challenges in COVID-19 Response Compared with 1918–19 Influenza Pandemic Response, New Zealand (Aotearoa)
Source: Emerg Infect Dis. 2023 Sep;29(9):1827–36. doi: 10.3201/eid2909.221265 (PMC10461674; doi:10.3201/eid2909.221265)
Supplement: Appendix — Additional information about improvements and persisting challenges in COVID-19 response compared to 1918 influenza pandemic response, New Zealand (Aotearoa) [file 22-1265-Techapp-s1.pdf]

Article DOI: https://doi.org/10.3201/eid2909.221265

EID cannot ensure accessibility for supplementary materials supplied by authors. Readers who have difficulty accessing supplementary content should contact the authors for assistance.

Improvements and Persisting Challenges in COVID-19 Response Compared to 1918-19 Influenza Pandemic Response, New Zealand (Aotearoa)

Appendix

Methods

We aimed to review and summarize relevant literature related to the two pandemics (1918–19 influenza pandemic and the first 3 years of the Covid-19 pandemic) in relation to NZ and where applicable, international literature. We conducted literature searches using online databases, such as Google Scholar, Embase, PubMed, NZ Government sources (as listed in the references), various media sources and gray literature, with searches undertaken between May and December 2022. We also drew upon our own expert knowledge with regards to both pandemics in NZ. Mortality data for both pandemics was sourced from a variety of sources including publicly available data, by previous research conducted by one of the authors *(1–5)* and provided directly from the NZ Ministry of Health in January 2023 for the purposes of this study *(6)*.

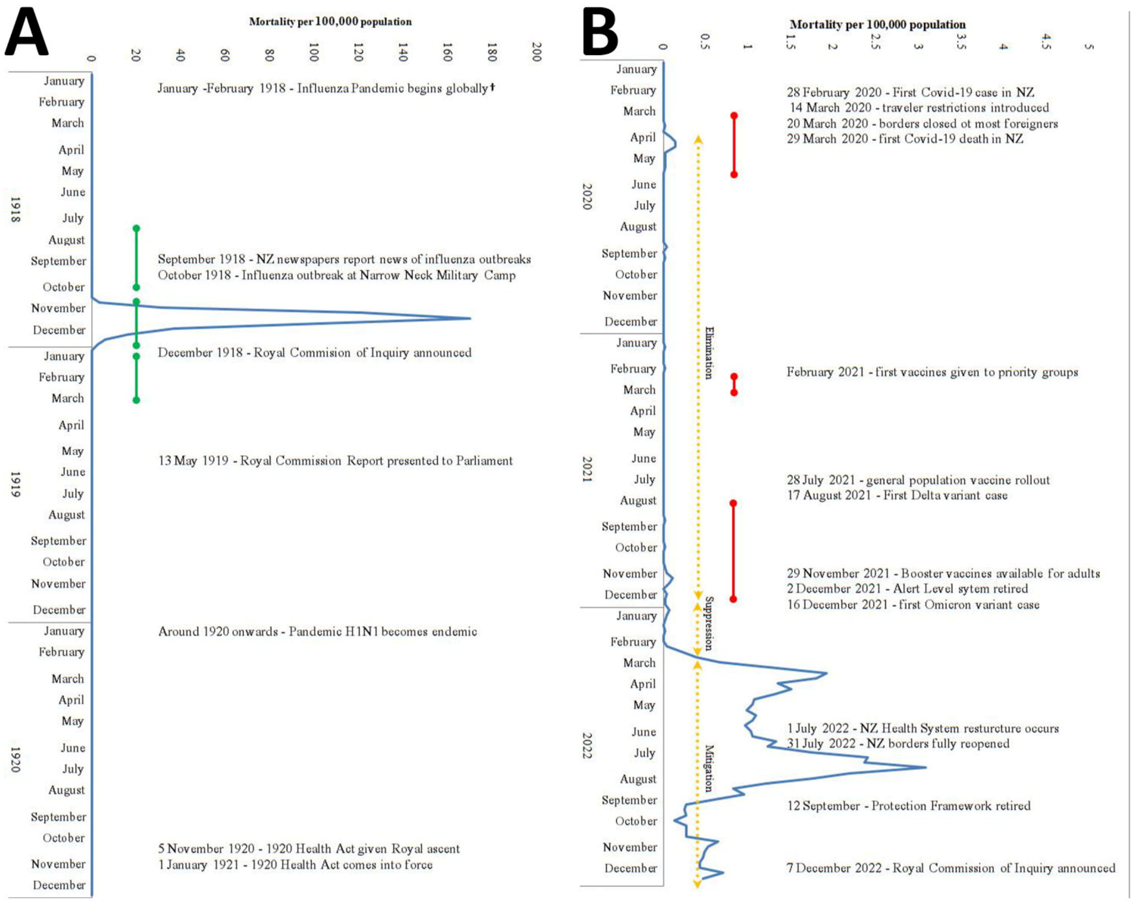

Appendix Figure 1. A) The 1918–1919 influenza pandemic mortality by week per 100,000 in New Zealand with pandemic waves and select pandemic related events. B) Covid-19 pandemic mortality by week per 100,000 in New Zealand, with corresponding lockdown periods and select pandemic related events. Green bars indicate three influenza pandemic waves of cases in NZ during 1918 and 1919: a first wave occurring in July-October 1918 with relatively low mortality, a more severe and lethal second wave between November-December 1918, and a relatively mild third wave in early 1919. Red lines indicate periods of national or local lockdowns in NZ during 2020 and 2021 - defined as Alert Levels 3 and 4 and used in various periods during 2020 and 2021. See Appendix Table 2 for further details of lockdowns in NZ. Yellow dotted lines and arrows indicate phases of responses: elimination (begins 25 March 11.59pm), followed by suppression (begins 2 December 2021 11.59pm), and finally mitigation (begins 16 February 2022 11.59pm). 1918 Influenza Pandemic mortality derived from several sources *(2,7,8)*, and population denominators (for the European population) estimated from population values between 1916 and 1921 in NZ *(3)*. Mortality totals include civilian deaths occurring in NZ along with identified military deaths occurring in the Southern Hemisphere during the pandemic period as previously described (note: there may be some minor overlap between civilian and military deaths) *(8)*. NZ military pandemic deaths occurring either ‘at sea’ or in the Northern Hemisphere are not included. Civilian deaths do not account for undocumented pandemic deaths occurring within the Māori population, so total civilian deaths are likely to be an underestimate. Covid-19 mortality, population denominators and details of lockdowns and select pandemic related events

derived from publicly available data from NZ's Ministry of Health *(5)*, Statistics New Zealand *(4)* and other sources *(9–11)*. Data extracted on 2 January 2023 and covers the period of February 2020 (when first case identified) to week ending 1 January 2023 (data given in weekly totals). †There is scientific debate as to whether influenza waves before 1918 constitute evidence of earlier pandemic waves *(12,13)*.

Appendix Table 1. Comparison of some of New Zealand's 1918–19 influenza pandemic and Covid-19 pandemic experiences and responses

Research Domain	1918–19 Influenza Pandemic	Covid-19 Pandemic - up to 31 December 2022
Epidemiology	• A total of 8,831 influenza pandemic related deaths among New Zealanders have been identified, however, it is estimated that the true number is more than 9,000 if undocumented deaths among Māori are considered *(1,2,14)*.	• As of the end of 2022, NZ reported a total of 2,115,241 cases of Covid-19 (confirmed and probable) *(5)*.
	• Morbidity figures are unavailable for NZ, although global estimates suggest a cumulative infection of 25%–50% *(15–17)*.	• There were 2,174 Covid-19 reported cases in 2020, 12,032 reported cases in 2021, and 2,101,035 cases reported in 2022. A total of 2385 Covid-19 deaths occurred in NZ up to the end of 2022 *(5)*.
	• Estimates of the reproduction number (R_0) in NZ range from 1.3 to 3.1 in military settings and 1.2 to 1.8 in community settings *(7,15,18)*.	• For the first two years of the pandemic, reverse transcription polymerase chain reaction (RT-PCR) was the standard method used for testing for Covid-19. In early 2022, rapid antigen tests (RAT) were made available to the public along with a reliance on the public to self-report RAT results to health authorities.
	• Mean generation time estimate 'as short as 3 d', and the proportion of asymptomatic cases estimated 'as large as 45%' *(7)*.	• Of the confirmed and probable reported cases in NZ up to the end of 2022, ~54.3% were among females and ~45.6% among males *(5)*.
	• Mortality rates showed a 'w' shaped distribution for age with young adults, in particular males, experiencing unexpectedly high mortality rates (combined with a higher risk in children under five years and increasing risk for 65+ age-groups) *(1,2,19–26)*. This distribution was consistent with many mortality patterns observed internationally *(27,28)*.	• Estimates of R_o (basic reproduction number) and R_{eff} (effective reproduction number) for Covid-19 vary depending on the variant and transmission dynamics. Globally, the original wild type strain is estimated to have a R_o = 2.79 *(29)*, whereas variants of concern are generally higher.
	• There was a temporary ban on publication of official mortality statistics in NZ *(2)*.	• In early 2020, before the Alert Level 4 in March 2020, the R_{eff} in NZ was estimated to be R_{eff} = 1.8, and reduced in the period after the lockdown to R_{eff} = 0.35 (R.N. Binny et al., unpub. data, https://www.medrxiv.org/content/10.1101/2020.08.10.20172320v1). During the Delta outbreak in August 2021 with an Alert Level 4 lockdown, the median effective R_{eff} reduced from R_{eff} = 6 to R_{eff} <1 (with a corresponding 90% reduction in transmission) (R.N. Binny et al., unpub. data). However, this initial reduction waned, and the median effective R_{eff} increased to 1.4 (90% confidence interval [CI] = 0.5 to 3.7). The first Omicron variant wave beginning in January 2022 (initially fueled by the BA.2 sub-lineage), the R_{eff} reached a peak in mid-February 2022 of >2 in NZ *(30)*.
Long-term effects of infection	• Longer-term effects of the influenza pandemic included life-course impacts for those exposed in utero: in the U.S., this birth cohort experienced markedly reduced educational attainment and increased disability rates compared with earlier and later cohorts *(31)*.	• During the pandemic, a post-Covid-19 syndrome known as '*long* Covid' has emerged *(32)*. Those with long Covid report lingering symptoms from their Covid-19 infection, such as fatigue, pain, shortness of breath, and 'brain fog'. There are also various forms of organ and tissue pathology following Covid-19 infection which manifest in various ways including excess mortality for at least a year following acute infection *(33)*.
	• Long-term effects following influenza pandemics were also recognized internationally *(34,35)*.<br>• A 2016 NZ study investigated the effect on lifespan from exposure to pandemic influenza, but found no statistically significance difference between an exposed and comparison cohort *(37)*.	• In late 2022, NZ's Ministry of Health produced clinical guidelines for the management of long Covid *(36)*.<br>• Internationally there is evidence for multisystem inflammatory syndrome in both adults (MIS-A) and children (MIS-C) which is an ongoing concern for NZ *(38–40)*.<br>• A comparison of Covid-19 with influenza (using international data) suggest that post infection sequelae are more common following Covid-19 infection *(41)*.
Virology	• The H1N1 virus is thought to have emerged from an avian reservoir as a spill over event.	• SARS-CoV-2 (the virus which causes Covid-19) is thought to have emerged from a bat reservoir to infect humans as a spill over event *(42)*. However, there is to date no clear consensus on this source for the pandemic.

Research Domain	1918–19 Influenza Pandemic	Covid-19 Pandemic - up to 31 December 2022
	• The influenza virus is generally spread through direct contact or through droplet dispersal (eg, from sneezing) *(43)*.	• SARS-CoV-2 has been found to be largely spread through respiratory particles (aerosols or and droplets), with aerosols increasingly recognized as the dominant mode of transmission; direct contact/ fomites may also aid virus spread *(44,45)*. The transmission risk is increased in indoor/crowded settings (particularly with poor ventilation), and when in close contact with an infected person *(46)*.
	• Secondary infections such as pneumonia (both viral and bacterial) are common, and account for a significant proportion of the final causes of death among influenza cases; as was the case for the 1918–19 influenza pandemic both globally and in NZ.	
Pandemic spread	• The 1918–19 influenza pandemic first spread in NZ between July and October in 1918 and this first wave was reported to be relatively mild. However, a substantially more lethal second wave occurred in November 1918 and spread to almost all parts of NZ.	• Covid-19 was first reported in NZ on 28 February 2020. Also in February 2020, New Zealanders in Wuhan, China were repatriated into a temporary isolation facility in Whangaparaoa *(47)*.
	• The second wave peaked in Auckland first, followed by the rest of the North Island, with the South Island peaking in mortality about a week later *(2)*.<br>• The impact of crowds for Armistice celebrations and other events such as Christchurch's *'race-carnival week',* have often been described as likely to have helped spread the virus from both within cities and then to more rural areas of NZ *(2,14,23,28)*.	• NZ borders were closed to non-citizens/residents in March 2020. Initial requirements were for self-isolation *(11,47)*.<br>• Managed Isolation and Quarantine (MIQ) facilities were introduced in April 2020 to accommodate all individuals returning to NZ (with few exemptions). The MIQ facilities helped to maintain the Covid-19 elimination strategy of the NZ Government for most of the first two years of the Covid-19 pandemic, albeit with some quarantine system failures *(48)*.
	• An earlier wave of the pandemic occurred in the East Coast and Poverty Bay-Wairoa regions, which has been described as possibly conveying immunity to some of the Māori population in these regions before the subsequent influenza waves *(49)*.	• An initial outbreak of Covid-19 peaked with 84 community cases on 25 March 2020 with cases throughout NZ. Smaller outbreaks occurred subsequently, for example in August 2020 *(50)*. In August 2021, a Delta variant outbreak was identified in Auckland with a first peak of 79 cases in the community on 26 August 2021, and a second peak of 226 cases in the community on 10 November 2021 *(5)*.
	• There are examples in NZ of successful measures to prevent outbreaks and spread (see below: ' Outbreaks and Land Borders') *(2,51)*.<br>• At a global level, the 1918–19 influenza pandemic occurred in three waves: a first 'spring' wave beginning in March 1918, a second 'fall' wave from September to November 1918, and a third wave in early 1919 *(52)*. NZ experienced the severe second wave and milder third wave.	• An Omicron wave with associated community transmission began in early 2022.<br>• At a global level, Covid-19 has occurred as a series of waves that have generally been dominated by successive variants. The NZ response greatly attenuated the waves experienced here, with relatively small outbreaks of the original variant in 2020 and the Delta variant in 2021. The Omicron variant waves in 2022 were large and comparable to that observed in other Asia-Pacific countries. At the time of writing, it was not clear whether Covid-19 can be considered to have become endemic in NZ (eg, since the waves have not fitted with any clear seasonal pattern).
	• The H1N1 virus became 'endemic' by around 1920 internationally, but continued to cause more severe influenza seasons for several years *(52)*.	
Outbreaks and local control measures	Local outbreaks in NZ are described for a variety of areas:<br>• The town of Nightcaps in Southland experienced one of the highest mortality rates in the European population, due to high morbidity (estimated at 80%) plus *'general unpreparedness of the community'* despite instructions from the Health Department to do so *(53)*. Over a two week period, of the population of 911 of Nightcaps and surrounding area, 41 individuals died, a mortality rate of 45 per 1,000 population.	• In NZ, multiple clusters of Covid-19 cases emerged following the first identified case in February 2020 *(54)*; including several aged residential care facilities, onboard ships, and various family/whānau gatherings *(5)*. As of the end of 2021, there were 24 clusters identified by the NZ Ministry of Health (defined as *'groups of ten or more people who likely caught the disease from one another'*), the largest being the *'August 2021 Community'* cluster in Auckland *(5)*. Since the arrival of Omicron in 2022 and widespread community transmission, clusters were no longer reported publicly in NZ.

Research Domain	1918–19 Influenza Pandemic	Covid-19 Pandemic - up to 31 December 2022
	• In the Coromandel Peninsula relatively intensive control measures were used, such as quarantining of ferry boat passengers and road closures around the Coromandel peninsula *(51,55)*. When an outbreak occurred among the Māori population in Manaia, the local chief [name not listed] worked with health officials to ensure isolation of infected cases. Food/medicine parcels were delivered every second day to those in household isolation in Manaia. In the town of Coromandel, the mortality rate for Europeans was found to be significantly lower than the rest of the peninsula and the rest of the District *(51)*. There was no evidence for a significant difference in Māori mortality rates.	• In March 2020, the NZ Government introduced the four-tiered Alert Level system *(9)*. At the higher levels of 3 and 4, there were restrictions of movement outside of regions and homes. Between 2020 and 2021, the Alert Level settings changed by region between the lowest at 1 up to 4, in response to the identification of community cases of Covid-19, meaning that travel was restricted to certain parts of NZ.
	• Temuka - with disease spread reportedly occurring via Armistice Day (11 November 1918) and Christchurch Races and Show Day (6–9 November 1918). The Māori population in this area were assigned to a separate temporary hospital with an assigned nurse. Separate health provision for the Māori population also occurred in other areas in NZ.	• Public health measures such as physical distancing, mass testing and face covering requirements were introduced in early 2020.
	• Nelson - one account gives an overview of cases in Nelson in one hospital and describes the isolation/quarantine measures used for both Nelson College and those in infected houses *(56)*.	• Between April and July 2021, a 'trans-Tasman' quarantine travel bubble between NZ and Australia was in place, however, this was suspended due to increasing community cases in Australia.
	• HMNZT Tahiti - an outbreak of influenza occurred onboard a NZ troopship, the HMNZT 107 Tahiti. This is described *as 'one of the worst ship outbreaks of pandemic influenza worldwide in 1918–19'* with a mortality rate of 68.9 persons per 1,000 population *(20)*. Factors such as crowding, poor ventilation and a younger age were associated with increased mortality risk. An exhibition in 2021–2022 at NZ's National Army Museum in Waiouru explored the *'fateful voyage'* of HMNZT Tahiti *(56)*.	• In January 2021, quarantine free travel with the Cook Islands opened, although testing requirements were put in place *(47)*.
	• Narrow Neck Military Camp in Auckland experienced an earlier influenza outbreak in October 1918 (with no deaths), and had a relatively lower mortality rate compared to other NZ military camps whose first exposure occurred during the November 1918 wave *(8)*. Narrow Neck had a mortality rate of 3.9 per 1,000 population, compared to Featherston Camp with 20.4 per 1,000, Awapuni Camp with 22.1 per 1,000, and Trentham Camp with 23.5 per 1,000.	• As part of border entry restrictions in 2020 and 2021, NZ identified other countries by their level of risk, with no travelers allowed to enter from those classified as high-risk. For example, in late 2021, with the emergence of the Omicron variant, several African nations were classified as high-risk (even when Omicron cases had been identified in other non-African nations, such as the United Kingdom) *(10)*.
	• Te Urewera 1918 - travel was restricted into Te Urewera region largely protecting the Tūhoe iwi *(57)*.	• Additional border entry requirements introduced in 2021 required full vaccination (two doses) of Covid-19, along with the Covid-19 testing requirements (PCR and/or rapid antigen tests) introduced in 2020.
		• On 2 December 2021, NZ moved away from the Alert Level System and the NZ Government implemented the Covid-19 Protection Framework, also known as the 'traffic light' settings. This framework placed emphasis on vaccination requirements for indoor environments and less on movement restrictions in NZ as was part of the earlier Alert Level System *(58)*.
		• The Covid-19 Protection Framework was retired on 12 September 2022. Most mask-wearing requirements, all vaccine mandates, and vaccination requirements for incoming travelers were removed. Self-isolation requirements for 7 d were retained only for positive Covid-19 cases *(59)*.
		• In mid-2022, a new Strategic Framework for Covid-19 Variants of Concerns was developed to describe potential future scenarios for the Covid-19 pandemic in NZ along with response measures *(60)*.
		• During the early phases of the pandemic when elimination and suppression strategies were being used, the regional borders in NZ were patrolled largely by

Research Domain	1918–19 Influenza Pandemic	Covid-19 Pandemic - up to 31 December 2022
		police-run check-points. Exemptions for crossing these borders were available, but were largely restricted to essential services and exceptional reasons such as attending funerals.<br>• There are also examples of land border restrictions run by some iwi during the pandemic. Examples include the Tai Tokerau Border Control in Northland *(61)*, specific holiday spot closures such as the Maitai Bay Campground on the Karikari Peninsula by Ngāti Kahu *(62,63)*, and Tūhoe in the Urewera area who set up road closures/checkpoints and testing centers *(64,65)*.
Impact on Māori	• The best available estimate of Māori mortality using registered deaths is 2,160; although this is likely an underestimate *(14)*.	• Māori had a disproportionately higher number of Covid-19 cases and higher risk for mortality compared to the European/Other ethnic grouping *(5,66,67)*.
	• A comparative analysis of three pandemics in NZ found that during the 1918–19 influenza pandemic, Māori mortality was 7.3 times higher among the civilian population and 2.3 times higher in the military population when compared to European populations in NZ *(68)*.	• Māori had a higher hospitalization risk following Covid-19 infection and possibly a longer length of stay in hospital compared to European/Other *(67,69)*
	• Theories for the differential mortality among the Māori population compared to the European population include the impact of higher rates of chronic disease (such as tuberculosis), relatively poorer access to health care/social support (partially due to a larger proportion living in rural settings), and potentially less exposure to early waves which may have conferred some form of immunity to the later more virulent pandemic waves *(2,14,26,49,68,70–76)*.	• Vaccine rates among Māori were proportionately lower than non- Māori *(5,77)*. Requests for a more equitable vaccine roll-out given the younger Māori age structure were made *(78)*, but differential prioritization based on age was not used in 2021. Second boosters were made available in mid-2022, with some eligibility based on age and ethnicity.
	• One study exploring specific mortality data for Māori found that as the Māori population in 1918–19 faced the high mortality rate among young adults, a resulting issue was the reduced numbers of caregivers within Māori villages *(73)*.	• A July 2021 study found that Māori had 2.5 times higher odds for being hospitalized (95% CI: 1.3 to 4.51) compared to non-Māori non-Pacific peoples *(69)*.
	• A ban on tangihanga (funeral rite) and associated travel was put in place in November 1918 *(2)*. This ban became a complete ban on public transport use by Māori. Travel was then only permitted for Māori who had been issued a permit. This affected the Member for Parliament (MP) for Northern Māori, Taurekareka (Tau) Henare who was initially refused travel from Auckland to Wellington (for parliamentary duties) until he obtained a permit *(2,79)*. One example is where Māori were banned from entering Whangarei, and then later requiring a health permit to enter *(2,80)*. In some places in NZ, the ban on travel for Māori expanded to unofficial bans on Māori entering business premises *(2)*, and other forms of discrimination *(81,82)*. The ban on tangi occurred along with the *'officials insistence on prompt burial of epidemic victims, many Māori flu victims were buried without ceremony in shallow graves close by the temporary hospital or whare where they happened to die' (2)*.	• Early modeling and analysis predicted that Covid-19 could have disproportionate negative effects on Māori compared to European/others). For example, infection fatality risks >50% higher for Māori (particularly when age and underlying health conditions were taken into account) *(83–85)*.
	• Examples of dedicated medical aid provided to Māori communities include military aid/provision *(86)*, relief workers to Māori settlements, influenza treatment pamphlets from the Health Department printed in Te Reo (Professor Geoffrey Rice describes how these pamphlets were distributed both slowly and erratically), various relief parties and volunteers, and separate hospitals for Māori patients *(2,87)*.	• At early stages and ongoing throughout the pandemic, there have been numerous calls for the incorporation of Te Tiriti o Waitangi principles *(88)* in the management of Covid-19 *(78,89,90)*. The NZ Government developed a Covid-19 Māori Health Protection plan which was informed by Te Tiriti o Waitangi *(91)*.<br><br>• During the first pandemic outbreak and among those in aged residential care, the rate of hospitalization was lower for both Māori (7.4% vs. 10.9%, p = 0.046) and European residents (7.4% vs. 10.9%, p = 0.046) compared to the

Research Domain	1918–19 Influenza Pandemic	Covid-19 Pandemic - up to 31 December 2022
		comparative period pre-Covid *(92)*. Māori residents also reported lower rates of loneliness compared to pre-Covid, while European residents *'reported more severe depressive symptoms'*.<br>• National Māori led support, health and information programs were developed. For example, Te Rōpū Whakakaupapa Urutā - National Māori Pandemic Group, iwi and Māori-health providers *(93,94)*.<br>• Nationwide restrictions and limitations on funerals/tangihanga periodically throughout the Covid-19 pandemic have had a negative impact on many New Zealanders. One article describes the impact of Covid-19 on tangihanga restrictions along with a description of how modern technology was used: *'Whānau Māori from across the country challenged to reimagine tangihanga and grieving during a global pandemic, highlighted the importance and therefore, the reliance of human beings on technology for connection' (95)*.
Risk factors for infection/ mortality and populations at increased risk	• The impact of poor-quality housing on mortality and morbidity from the 1918–19 influenza pandemic has been evaluated for several communities in NZ. For example, poor housing and crowded conditions have been shown to have negatively affected outcomes in Wellington, Christchurch and among the Māori community *(2,28,49,75,96–98)*. The *'slum'* conditions of many urban areas in NZ in 1918–19 was widely reported in newspapers discussing the pandemic at the time *(99)*, and a more recent study in 2018 found evidence for socio-economic gradients for mortality in NZ *(100)*.	• There was a higher risk for Covid-19 mortality among Pacific peoples when compared to European/Other group in analysis of data up to August 2022 *(66)*.
	• One study assessed the effect of rurality on mortality during the 1918–19 pandemic and found that individuals in small towns in NZ had a higher mortality rate than large towns and cities, possibly due to less previous exposure or perhaps less access to health care compared to cities *(101)*. Recent studies assessing NZ military populations found a variety of factors for mortality risk, with a rural background associated with increased mortality risk among Māori/Pacific personnel *(74)*, increased mortality risk with rural background among all military personnel in WW1 *(8)* and a larger chest size (as measured as part of uniform requirements and the increased mortality risk theorized as possibly an indicator of larger bodies and therefore an increased chance of a cytokine storm response *(102)*. Personnel based in the Northern Hemisphere during the pandemic had no difference in mortality risk based on rurality background. But for those located in the Southern Hemisphere during the pandemic (largely in NZ military camps), there was a increased mortality risk if from a large town or rural background (compared to a city background) *(1)*.	• Also, a higher hospitalization risk for Pacific people following Covid-19 infection and possibly a longer length of stay in hospital compared to European/Other *(69)*.
		• Early modeling and analysis predicted that Covid-19 could have disproportionate negative effects on Pacific peoples compared to European/others), particularly when age and underlying health conditions were taken into account *(83,85)*.<br>• Pacific peoples had 3 times higher odds for being hospitalized from Covid-19 (95% CI: 1.75 to 5.33) compared to non-Māori non-Pacific peoples *(69)*.<br>• Vaccine rates among Pacific peoples were overall proportionately lower than non-Māori-non-Pacific, however this difference varied by age group *(5)*.<br>• Higher rates of severe depression were found among European residents in aged residential care compared to the pre-Covid period, while the authors

Research Domain	1918–19 Influenza Pandemic	Covid-19 Pandemic - up to 31 December 2022
		reported that they *'did not find any immediate negative impact of the first wave of Covid-19, which included a nationwide lockdown, on the health and psychosocial well-being among older Māori and Pacific Peoples living in ARC [aged-residential care]' (92)*.<br>• The Covid vaccination program in NZ began by targeting particular groups at increased risk of being infected, such as MIQ workers and their families, those living in high-risk settings such as aged-care facilities and other priority groups (for example, Māori and Pacific providers, those living in Counties Manukau DHB and over 65 y of age or with an underlying condition) *(103)*. Targeted vaccine delivery programs were also incorporated into the vaccine roll-out, such as by working with Māori health providers.<br>• Concern has been raised regarding the inequitable burden from Covid-19 (both in terms of infection but also the effects of management and economic impacts) on particular groups in NZ, such as Māori and Pacific peoples communities *(90)*.
Impact on children and pregnant women	• Pandemic mortality among children varied with age, with 0–4 y olds accounting for 5% of all deaths in NZ, 5–9 y olds 0.9%, 10–14 y olds 1.2% and 15 to 19 y olds 4.4%, with an overall total of 11.5% of all deaths from the pandemic in NZ *(2,73,97)*.	• As of the end of 2022, Covid cases among children aged 0 to 19 y account for 22.9% of total reported cases in NZ *(5)*.
	• During the second wave, many schools were closed, exams cancelled (with final marks assigned based on school records), and universities initially stayed open until the death of a student from the pandemic *(2)*.	• Children are generally considered to typically experience less severe illness from Covid-19 than adults *(104–106)*.
	• A thesis examining the pandemics impact on children in NZ describes how children's *'understanding of the epidemic were often shaped by family members' participation in flu-relief work' (107)*. During this period, children were impacted by both the pandemic and the war.	• During periods of lockdowns (Alert Levels 3 and 4), schools and universities were generally required to close, unless needed to provide education for children of essential workers. Most education during these periods was provided digitally and at-home mainly by parents/caregivers. Notably, during the Omicron outbreak from early 2022, the Ministry of Education directed schools to stay open, although some schools chose online learning or were compelled to do so due to illness among students and staff.
	• Children were involved with the relief effort, such as delivering groceries and medical supplies throughout their communities. For example, the senior students at Auckland Marist Brothers School provided assistance by being messengers and also orderlies in the influenza wards *(2)*.	• Exposure to infection in schools appears to have been a significant factor in transmission, and there have been calls for improvements to ventilation within schools (with some subsequent action by the Ministry of Education), and also for the NZ Government to consider a whānau (family)-centered approach to protecting children *(108)*, rather than a school or child-centered approach where school closures are seen as a last resort for managing Covid *(109)*. One study found that many NZ parents and caregivers expressed concern about children getting infected with Covid-19 while in the school-setting *(106)*. National Covid-19 rates show that the highest rate by occupation is in school teachers *(110)*.
	• Christ College in Christchurch experienced an early infection wave in September/October 1918, with some evidence to suggest that it may have conferred some immunity to the later more lethal November 1918 wave *(2)*, similar to the effect observed at Narrow Neck Military Camp *(8)*.	• Criticism has been made about the lack of comprehensive guidelines for protecting children (and their whanau) from Covid throughout the pandemic *(108,111)*, and during pregnancy *(112)*, although the Ministry of Health provides some guidance on vaccination and managing Covid-19 while pregnant *(113)*.
	• Children whose parents were unwell were in some cases put into temporary care *(2)*.	• One study evaluated the impact of Covid-19 lockdowns in terms of health and economic impacts among Pacific children and their families. The authors suggest that there were differential impacts on different cohorts of children, in particular Pacific children *(114)*.

Research Domain	1918–19 Influenza Pandemic	Covid-19 Pandemic - up to 31 December 2022
	• A 2015 review found that during the 1918–19 influenza pandemic, a lot of responsibilities shifted onto children as their parents were incapacitated, thus becoming primary caregivers *(115)*.	• A surveillance study undertaken in 2020 found evidence for a detrimental impact of Covid lockdowns particularly for newborns in terms of delayed care for non-Covid-19 related conditions. This study described the findings as showing the unintentional harms for children due to the Covid-19 lockdowns *(105)*.
	• The high mortality rate experienced by young adults resulted in issues with lack of caregivers for children in Māori villages *(73)*.	• The partial reopening of NZ's border restrictions to Australia in April 2021 was associated with a substantial increase in the incidence of children infected and hospitalized with respiratory syncytial virus (RSV) *(116)*.
	• One study explored the impact of the pandemic on pregnancy, reporting that pregnancy outcomes were adversely affected by infection with many pregnant patients *"aborted, or miscarried and did badly" (117)*. And one study reported a decrease in fertility among Māori women following the pandemic *(49)*.	• Concerns have been raised during the pandemic about teenagers not returning/finishing secondary education after lockdowns, with some teenagers taking up paid employment to support families *(118)*.
	• In 1918 and 1919 there was a sudden decrease in NZ's annual birth rate compared to 1917 *(3,28,119)*, which was possibly related to the relationship between influenza infections and stillbirth and fetal loss. A 2019 study estimated that in both 1918 and 1919 there were fewer births compared to 1917: for Māori, a reduction in birth rate per 1,000 population of 6.7% and 19.8% respectively, and for non-Māori, a reduction in birth rates per 1,000 population of 8.8% and 16.6% respectively *(119)*. Internationally, there is also evidence on negative impacts of influenza infection on birth rates *(120,121)*.	
Economic impacts	• Professor Rice describes how the cost of the pandemic *'in monetary terms was very considerable' (2)*. The costs to the NZ Health Department were substantial and included those of setting up temporary hospitals, epidemic relief work, and salaries contributing to the bulk of the direct costs. Alongside this, there was the cost of the Influenza Commission in 1919, and benefits for widows increased (along with the substantial increase in war widows due to WW1).	• In 2020, the NZ Government implemented a program to financially assist businesses and employees affected by the pandemic and related lockdowns, resulting in less economic harm from the pandemic compared to many other OECD countries *(122)*, with evidence this protection continued into mid-2021, also with an increase in unemployment which was less than the OECD average (N. Wilson et al., unpub. data, https://www.medrxiv.org/content/10.1101/2021.06.25.21259556v1). The international tourism sector and the education sector involved with overseas students were particularly severely impacted by the border restrictions.
	• There were issues with the inability for payment from the Government for services provided during the pandemic by individuals and businesses. One approach to handle the ill-feeling was to ask claimants to consider some of their services as a donation *(2,123)*.	• In December 2021, the NZ Government stated that GDP declined less than expected in the September 2021 quarter *(124)*.
	• Coastal shipping came to a *'complete standstill'* during the pandemic resulting in a shortage of essentials *(2)*. However, Professor Rice suggests that given that the pandemic was over so quickly, that there is no evidence of a significant impact on businesses, such as from closures.	• A March 2022 report from the NZ Treasury suggests that economic activity losses experienced in September 2021 had recovered substantially, although the effect of the war in Ukraine from February 2022 had affected market volatility *(125)*.
Response strategy	• The Department of Public Health was formed in 1903 in response to concerns about the potential arrival of plague in 1900.	• NZ produced its first pandemic influenza plan in 2002, which it revised periodically. The second edition was published in 2017 and was current at the emergence of Covid-19 (and remains so). It is based on a hypothetical influenza pandemic and predominantly takes a mitigation strategy *(126)*. This was the approach that the NZ Government seemed to take at the very start of the Covid-19 pandemic, before more clearly adopting an elimination strategy.
	• However, pandemic planning was not a well-established activity of NZ Governments until after the 1918–19 influenza pandemic.	• In response to the Covid-19 pandemic NZ effectively switched to an elimination strategy in March 2020 *(127,128)*. It subsequently shifted to

Research Domain	1918–19 Influenza Pandemic	Covid-19 Pandemic - up to 31 December 2022
		suppression during the Delta outbreak in 2021 *(129)*, and to a mitigation strategy during 2022 *(58,59)*.<br>• Implementing an elimination response strategy resulted in rapid end to the first outbreak of Covid-19 infection followed by a prolonged period with very little transmission *(130,131)*.<br>• Elimination and tight suppression were widely used response strategies in the Asia Pacific Region, though not always described explicitly *(132,133)*.<br>• Subsequent analysis provides support for the value of a strong strategic response to emerging infectious diseases with pandemic potential, particularly the value of an elimination strategy if certain conditions are met *(133–135)*.
Health system impacts	Multiple authors have evaluated the impact of the 1918–19 influenza pandemic on both the workforce and the health system as a whole:<br>• One substantial issue occurring in 1918 was that a significant proportion of the health workforce was absent due to war service or after falling ill with influenza themselves *(136,137)*. Furthermore, the Health Minister at the time, Mr GW Russell, was also responsible for multiple ministerial portfolios.	• The impact of Covid-19 management measures has been noted as impacting on management and treatment of other health conditions, such as acute coronary syndrome (although with a noted reduction of hospitalizations during lockdowns) *(138)*, and cancer diagnosis with delayed diagnosis during lockdown periods largely mitigated by *'catch-up'* activities post lockdowns *(139)*.
	• Pandemic mortality rates for medical practitioners and nurses were also notably high and nearly as high as for military personnel in the military camps *(2)*.<br>• Temporary hospitals were used to manage pandemic cases *(2,21,96)*.	• In April 2021, it was announced that the NZ Health System would be restructured, following 2019 recommendations from a review of the health and disability system *(140,141)*.<br>• The role of the Minister for Health was separated from the Covid-19 Response Ministerial portfolio in November 2020, with the Hon Chris Hipkins being appointed the first Minister for Covid-19 Response and the Hon Andrew Little taking up the role of Minister of Health.
	• A review of the nursing response in NZ found reports that in some places, the nursing ratio was 70–80 patients per nurse *(142)*.	• Vaccine mandates for certain work places such as health and education resulted in some redundancies, although financial support was made available for those who lost their jobs *(143)*.
	• The limitations of the NZ Health Department response were well documented, and were a substantial reason for the 1919 Royal Commission Report on the pandemic, which in turn led to the 1920 Health Act and the restructure of the NZ health system *(98,144–146)*. The Commission found that the NZ Health Department was unprepared for the pandemic emergency *(2)*. Professor Rice describes how even though the Commission was assembled quickly, there were aspects that were lacking in the report *(14)*. For example, there was little discussion of the impact of the pandemic on the Māori population.	• A Royal Commission of Inquiry was announced in December 2022 with the scope aimed at exploring the aspects of the NZ Covid-19 response that could be used to prepare for any future pandemics - for example, various legislative, regulatory and operational settings along with decision-making structures and *'consideration of the interests of Māori in the context of a pandemic, consistent with the Te Tiriti o Waitangi relationship' (147,148)*. Several aspects of the pandemic were deemed outside of the scope of the Inquiry, such as some epidemiologic features of the virus, strategies/measures *'devised in response to Covid-19'*, the health system reforms, vaccine efficacy, private sector response, response/adaption's in court proceedings, Reserve Bank monetary policies, and conduct of the general election during the Covid-19 pandemic *(148)*.
Vaccinations	• As the 1918–19 influenza pandemic was spreading, various vaccination studies were conducted worldwide, with some specifically among the NZEF located in Europe *(22,149–151)*. Some of these studies, when epidemiologically assessed using more modern statistical techniques, and despite the various methodological issues, suggest '*that whole-cell inactivated pneumococcal vaccines* [as used in 1918–19] *may confer cross-protection to multiple pneumococcal serotypes and that bacterial vaccines may play a role in preventing influenza-associated pneumonia' (152)*.	• At the beginning of the Covid-19 pandemic, there was no vaccine available, although clinical trials began in 2020.
	• In NZ, newspapers presented a mixed perception of the value of vaccination for the general public during the 1918–19 influenza pandemic,	• The Pfizer-BioNTech mRNA Covid-19 vaccine was approved for use among targeted groups in NZ from February 2021, with general roll-out for the wider

Research Domain	1918–19 Influenza Pandemic	Covid-19 Pandemic - up to 31 December 2022
	building upon the various commentary on the mass-vaccination of the NZEF in previous years *(137,153)*. For example, a 1918 headline in the Nelson Evening Mail reports '*evidence is pouring in from doctors of the efficacy of influenza inoculation for other disease of chronic types, such as rheumatism, neuritis, sclerosis, catarrh, neuralgia and asthma' (154)*, while a 1919 headline in the NZ Herald states '*'Epidemic commission. Medical Association View. Brought from overseas inoculation and masks. Their value doubted' (145)*. These conflicting headlines, occurred throughout and after the pandemic, with the efficacy of the vaccines used being raised and evaluated during the NZ Influenza Commission in 1919.	population from July 2021 onwards, with children given approval later (12+ years from August 2021 and 5+ years in January 2022) *(103)*.
	• Controversy and hesitancy for vaccination to various infectious diseases still persists into more modern times *(155)*, and is an ongoing cause for concern as vaccination rates fall in many countries.	• The roll-out of NZ's largest mass vaccination program was mostly successful with large sections of NZ's population receiving between one to four doses by early 2022.<br>• There were requests in 2021 for a more equitable Covid-19 vaccine roll-out, for example, taking into account the younger Māori age structure *(78)*.<br>• Among children, vaccine uptake has been particularly low with only ~27.8% of those aged 5 to 11 by the end of 2022 having received two vaccine doses (with boosters generally not available for children under 16 y of age) (*3*).<br>• The roll-out was also introduced alongside various vaccine mandates in late 2021 for large sections of the population, and was integrated into the revised Covid-19 Protection Framework ('traffic-light' system) which superseded the original Alert Level System *(58)*. Vaccine mandates were removed in September 2022 when the Framework was retired *(59)*.<br>• Throughout the Covid-19 vaccine program there was some public hesitancy and opposition to both receiving a vaccine or being required to be vaccinated to continue employment in certain occupations, to access education, or to gain access to various premises *(156)*. For example, the mandate for NZ Defense Force staff and NZ Police staff to be fully vaccinated (two doses) was challenged successfully in the High Court in February 2022 (although this decision was appealed by the Government) *(157,158)*.<br>• There was a four-week long protest outside the NZ Parliament in Wellington in February/March 2022 with anti-mandate and anti-vaccine rhetoric being conspicuous concerns (albeit mixed with a wide-array of other themes).<br>• At the same time, childhood vaccination levels for other infectious diseases have declined in NZ, particularly for Māori raising concerns for potential outbreaks of other vaccine-preventable diseases, and exacerbating existing health disparities.<br>• Vaccine hesitancy is not a new phenomenon, however the Covid-19 pandemic has brought this issue into the forefront of public attention.

Details of the period after the 1918–19 influenza pandemic in NZ

The period both during and following the 1918–19 influenza pandemic was a uniquely difficult time for New Zealanders: 'Out of the blue, just as the Great War [WW1] was at last coming to an end, communities and families were suddenly confronted with a life and death struggle over which they seemed to have very little control' *(2)*. This collective trauma led to a period described as a 'reckoning' *(2)*. There were many questions to be answered about the handling of the pandemic, but also a desire to learn from the experience. Hence, the establishment of the 1919 Royal Commission on the pandemic *(2)*. This inquiry led to legislative changes such as the 1920 Health Act, substantial changes to the health system, establishing the Public Health Department, and requirements for authorities to improve public health through intervention in key areas, for example, sanitation and housing *(2,28)*.

Following the 1918–19 influenza pandemic, there were a further three influenza pandemics in 1957, 1968 and in 2009. While the 1918–19 influenza pandemic had the greatest mortality both in NZ and internationally, the other three pandemics still had significant impact, particularly when considering ethnic gradients. In NZ, the mortality rates for the Māori population was found to be higher than non-Māori in all three pandemics, although with evidence of some decline in the extent of this difference by 2009 *(66)*.

Later responses to the 1918–19 influenza pandemic include a 2002 formal apology to Samoa by then Prime Minister Helen Clark, for NZ's 'inept and incompetent' administration leading to the spread of influenza throughout the Pacific by the NZ ship SS Talune *(159)*. This spread led to Western Samoa losing around 25% of its adult population due to the pandemic, one of the worst mortality rates globally *(2)*.

A 2017 study surveyed the memorials in NZ for the 1918–19 influenza pandemic finding only seven publicly accessible memorials, with 11 in private settings (Appendix Figure 2), compared to the 941 memorials in NZ for WW1/WW2 *(160)*. The lack of pandemic-related memorials could be due to a range of factors, such as the timing so soon after WW1. The study authors recommended how memorialization could be improved along with public education and support for future pandemic planning. Further recognition of this pandemic included a NZ national memorial plaque for the 1918–19 influenza pandemic. The plaque was unveiled in November 2019 at the Pukeahu National War Memorial Park in the

capital city, Wellington (Appendix Figure 3), which, ironically, was likely to have coincided with the first emergence of Covid-19 in China.

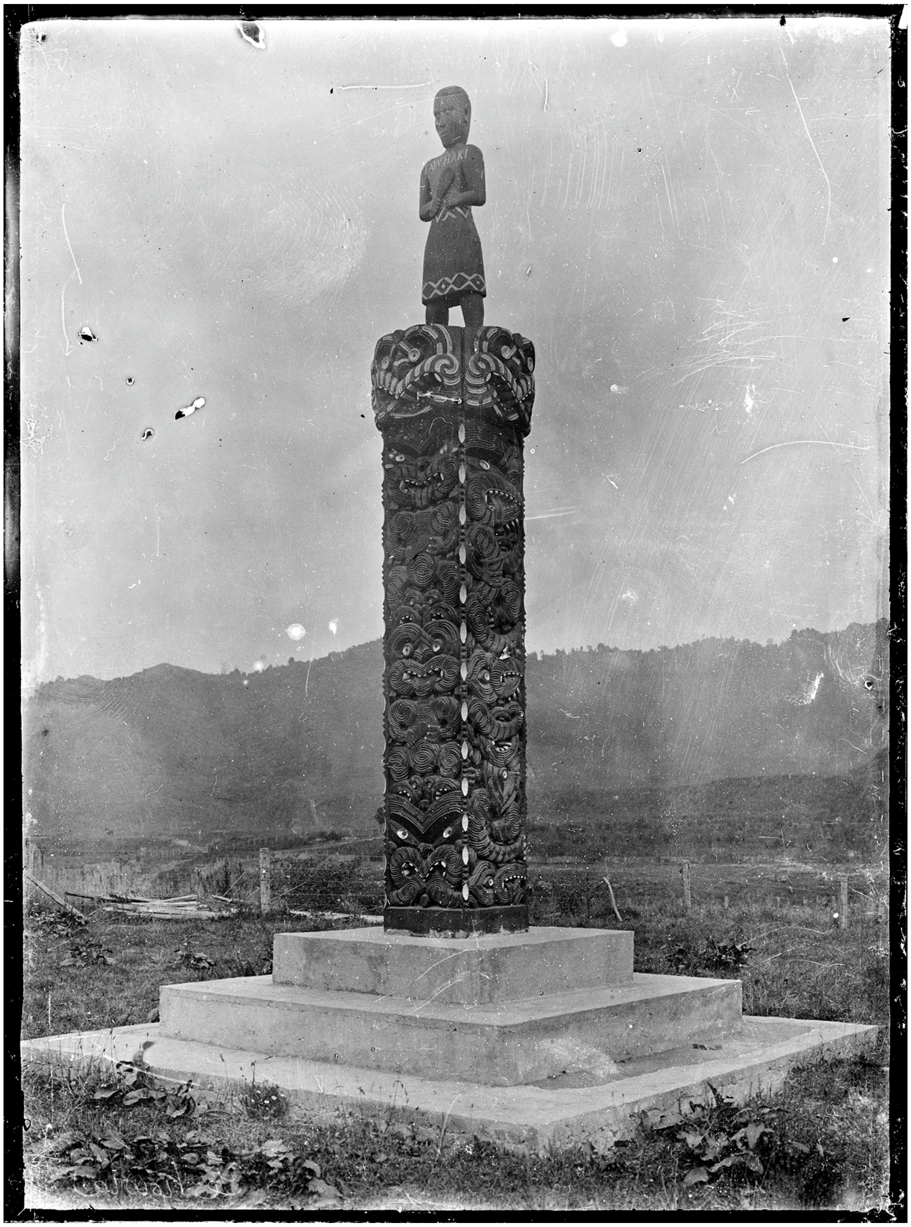

Appendix Figure 2. Carved wooden Māori cenotaph at Te Koura Marae, in memory of those who died in the influenza pandemic. Photographed in 1920 by Albert Percy Godber. Cenotaph designed and carved by Tene Waitere of Ngati Tarawahi, APG-0786–1/2-G, Editor. 1920: Alexander Turnbull Library, Wellington, New Zealand

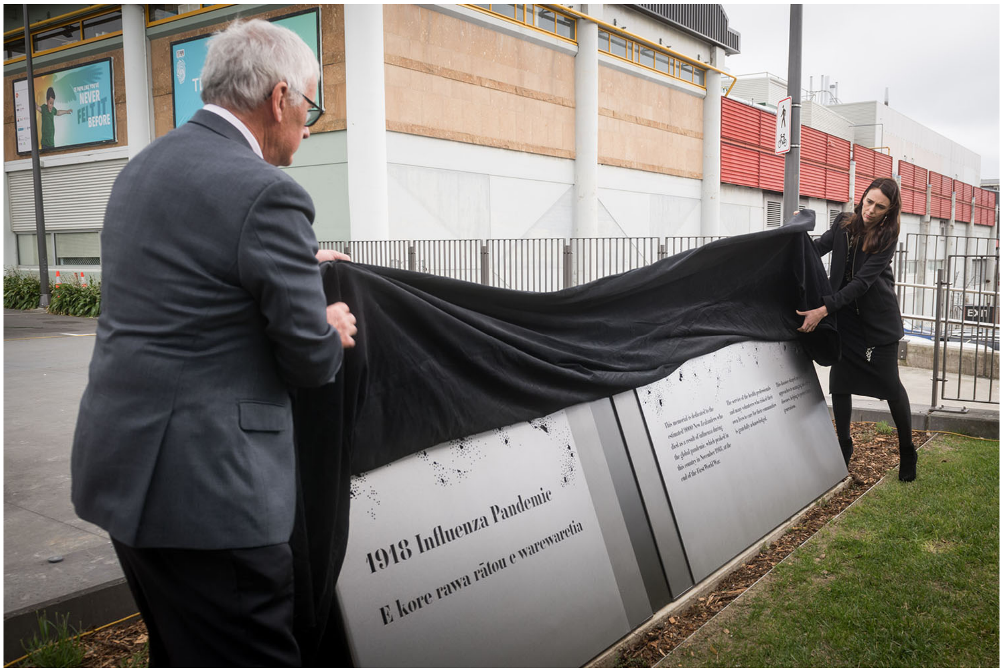

Appendix Figure 3. Geoffrey Rice, Emeritus Professor of History, University of Canterbury, and former Prime Minister Rt Hon Jacinda Ardern unveil 1918 Influenza Pandemic Memorial Plaque at Pukeahu. Photographed in 2019 by Mark Tantrum Photography. Available from Ministry for Culture and Heritage - Manatū Taonga. https://mch.govt.nz/significant-sites/1918-influenza-pandemic-memorial-plaque

Appendix Table 2. COVID-19 Alert Levels in NZ throughout 2020 and 2021*

Year	Date	Covid-19 Alert Level
2020	23 March	Level 3—all of NZ
	25 March	at 11.59pm—Level 4—all of NZ—Start of Elimination Strategy.
	27 April	Level 3—all NZ
	13 May	Level 2—all of NZ
	8 June	Level 1—all of NZ
	12 August	Level 3 in Auckland.
	30 August	Level 2 in Auckland (with travel and gathering restrictions). Rest of NZ remains at Level 2.
	21 September	All regions (except Auckland) at Level 1.
	23 September	Level 2 (without travel and gathering restrictions) in Auckland
	7 October	Level 1—all of NZ
2021	14 February	Level 3 in Auckland. Level 2 rest of NZ
	17 February	Level 1—all of NZ
	28 February	Level 3 in Auckland. Level 2 rest of NZ
	7 March	Level 2 in Auckland. Rest of NZ remains at Level 1.
	12 March	Level 1—all of NZ
	23 June	Level 2 in Wellington. Level 1 rest of NZ
	29 June	Level 1—all of NZ
	17 August	Level 4—all of NZ
	31 August	Level 4 in Auckland and Northland. Rest of NZ at Level 3.
	2 September	Level 4 remains in Auckland. Northland and rest of NZ at Level 3.
	7 September	Level 4 remains in Auckland. Rest of NZ at Level 2.
	21 September	Level 3 in Auckland and Upper Hauraki. Rest of NZ at Level 2.
	25 September	Level 3 remains in Auckland. Rest of NZ at Level 2.
	3 October	Level 3 remains in Auckland, and applied to various locations in the North Island. Rest of NZ at Level 2.
	5 October	Various locations in North Island remain at Level 3. Auckland and the rest of NZ at Level 2.
	7 October	Level 3 extended to wider Waikato in the North Island. Auckland at Level 3. Rest of NZ at Level 2.
	8 October	Level 3 extended to Northland.
	19 October	Northland at Level 2. Auckland and parts of Waikato at Level 3. Rest of NZ at Level 2.

Year	Date	Covid-19 Alert Level
	2 November	Upper Northland at Level 3. Auckland and parts of Waikato at Level 3. rest of NZ at Level 2.
	9 November	Level 2 in Auckland. Parts of Waikato and Upper Northland at Level 3. Rest of NZ at Level 2.
	11 November	Upper Northland at Level 2. Parts of Waikato and Upper Northland at Level 3. Rest of NZ at Level 2.
	16 November	Part of Waikato at Level 2. Auckland at Level 3. Rest of NZ at Level 2.
	2 December	At 11.59pm—COVID-19 Alert Level System retired and Covid-19 Protection Framework introduced. Start of Suppression Strategy.
2022	16 February	At 11.59pm—Official shift from identifying all Covid-19 cases as part of Phased reduction of protective measures. Start of Mitigation Strategy.
	12 September	Covid-19 Protection Framework retired.

*Alert Levels 1 (prepare) to 2 (reduce) = Non-lockdown rules with various public health restrictions/protections in place, such as mask-wearing, contact tracing and border entry restrictions in place (*9–11*). Alert Levels 3 (restrict) to 4 (eliminate) = Lockdowns in place (either nationally or locally) with heavy restrictions such as for travel, crossing borders, mass gathering cancellations and educational facilities closed (*9–11*).

References

1. Summers JA. The burden and risk factors for death from the 1918–19 influenza pandemic amongst the New Zealand military forces of World War One [thesis]. 2013, University of Otago.

2. Rice G. Black November: the 1918 influenza pandemic in New Zealand. Christchurch: Canterbury University Press; 2005.

3. New Zealand Department of Statistics,. The New Zealand official yearbook, 1924 [cited 2023 Jan 2]. https://www3.stats.govt.nz/New_Zealand_Official_Yearbooks/1924/NZOYB_1924.html

4. Statistics New Zealand, Tatauranga Aotearoa. Population. 2023 [cited 2023 Jan 4]. https://www.stats.govt.nz/topics/population

5. Ministry of Health–Manatū Hauora.. COVID-19 data and statistics. 2023 [cited 2023 Jan 4]. https://www.health.govt.nz/covid-19-novel-coronavirus/covid-19-data-and-statistics

6. Ministry of Health–Manatū Hauora. Covid-19 Mortality Data. New Zealand Government–Te Kāwanatanga o Aotearoa, Editor. 2023: Wellington, New Zealand.

7. Nishiura H, Wilson N. Transmission dynamics of the 1918 influenza pandemic in New Zealand: analyses of national and city data. N Z Med J. 2009;122:81–6. PubMed

8. Summers JA, Shanks GD, Baker MG, Wilson N. Severe impact of the 1918–19 pandemic influenza in a national military force. N Z Med J. 2013;126:36–47. PubMed

9. Unite against COVID-19. History of the COVID-19 alert system: about our COVID-19 response. 2022 [cited 2023 Jan 2]. https://covid19.govt.nz/about-our-covid-19-response/history-of-the-covid-19-alert-system

10. New Zealand Doctor. Timeline—coronavirus—COVID-19. 2022 [cited 2022 Dec 29]. https://www.nzdoctor.co.nz/timeline-coronavirus

11. Public Policy Institute. COVID-19 timeline: tracking key events in NZ and international milestones [cited 2023 Jan 2]. https://www.policycommons.ac.nz/covid-19-policy-resources/covid-19-timeline

12. Barry JM. The site of origin of the 1918 influenza pandemic and its public health implications. J Transl Med. 2004;2:3. PubMed https://doi.org/10.1186/1479-5876-2-3

13. Oxford JS. The so-called Great Spanish Influenza Pandemic of 1918 may have originated in France in 1916. Philos Trans R Soc Lond B Biol Sci. 2001;356:1857–9. PubMed https://doi.org/10.1098/rstb.2001.1012

14. Rice GW. Black flu 1918: the story of New Zealand's worst public health disaster. Christchurch: Canterbury University Press; 2017.

15. Wilson N, Summers JA, Baker MG. The 2009 influenza pandemic: a review of the strengths ;and weaknesses of the health sector response in New Zealand. N Z Med J. 2012;125:54–66. PubMed

16. Crosby AW. America's forgotten pandemic: the influenza of 1918. New York: Cambridge University Press; 2003.

17. Frost W. Statistics of influenza morbidity. Public Health Rep. 1920;35:584–97. https://doi.org/10.2307/4575511

18. Sertsou G, Wilson N, Baker M, Nelson P, Roberts MG. Key transmission parameters of an institutional outbreak during the 1918 influenza pandemic estimated by mathematical modelling. Theor Biol Med Model. 2006;3:38–38. PubMed https://doi.org/10.1186/1742-4682-3-38

19. Wilson N, Oliver J, Rice G, Summers JA, Baker MG, Waller M, et al. Age-specific mortality during the 1918-19 influenza pandemic and possible relationship to the 1889–92 influenza pandemic. J Infect Dis. 2014;210:993–5. PubMed https://doi.org/10.1093/infdis/jiu191

20. Summers JA, Wilson N, Baker MG, Shanks GD. Mortality risk factors for pandemic influenza on New Zealand troop ship, 1918. Emerg Infect Dis. 2010;16:1931–7. PubMed https://doi.org/10.3201/eid1612.100429

21. Summers JA, Wilson N, Baker MG, Gottfredsson M. The influenza pandemic of 1918–1919 in two remote island nations: Iceland and New Zealand. N Z Med J. 2013;126:74–80. PubMed

22. Champtaloup ST. A study of the influenza epidemic in New Zealand 1918: epidemiology, administration and bacteriology, with a report on the pathology of twenty-five fatal cases [dissertation]. 1920, University of Edinburgh.

23. Maclean FS. Challenge for health: a history of public health in New Zealand. Med J Aust. 1964;2.

24. Wright-St Clair RE. Influenza in New Zealand and the doctors who died from it. N Z Med J. 1983;96:765–8. PubMed

25. Rice GW. Influenza in New Zealand before 1918: a preliminary report. Am J Epidemiol. 2018;187:2524–9. PubMed https://doi.org/10.1093/aje/kwy180

26. Rice GW. 'That terrible time': reflections on the 1918 influenza pandemic in New Zealand. N Z Med J. 2018;131:6–8. PubMed

27. Johnson NP, Mueller J. Updating the accounts: global mortality of the 1918–1920 "Spanish" influenza pandemic. Bull Hist Med. 2002;76:105–15. PubMed https://doi.org/10.1353/bhm.2002.0022

28. Summers JA, Baker M, Wilson N. New Zealand's experience of the 1918–19 influenza pandemic: a systematic review after 100 years. N Z Med J. 2018;131:54–69. PubMed

29. Liu Y, Rocklöv J. The reproductive number of the Delta variant of SARS-CoV-2 is far higher compared to the ancestral SARS-CoV-2 virus. J Travel Med. 2021;28: taab124.

30. Abbott S, Hellewell J, Thompson RN, Sherratt K, Gibbs HP, Bosse NI, et al. Estimates for New Zealand. 2022 [cited 2023 Jan 4]. https://epiforecasts.io/covid/posts/national/new-zealand

31. Almond D. Is the 1918 influenza pandemic over? Long-term effects of in utero influenza exposure in the post-1940 U.S. population. Polit Econ. 2006;114:672–712. https://doi.org/10.1086/507154

32. Davis HE, McCorkell L, Vogel JM, Topol EJ. Long COVID: major findings, mechanisms and recommendations. Nat Rev Microbiol. 2023;21:133–46. https://doi.org/10.1038/s41579-022-00846-2 **PMID 36639608**

33. Wang W, Wang CY, Wang SI, Wei JC. Long-term cardiovascular outcomes in COVID-19 survivors among non-vaccinated population: a retrospective cohort study from the TriNetX US collaborative networks. EClinicalMedicine. 2022;53:101619. PubMed https://doi.org/10.1016/j.eclinm.2022.101619

34. Honigsbaum M, Krishnan L. Taking pandemic sequelae seriously: from the Russian influenza to COVID-19 long-haulers. Lancet. 2020;396:1389–91. PubMed https://doi.org/10.1016/S0140-6736(20)32134-6

35. Schroeder M, Lazarakis S, Mancy R, Angelopoulos K. An extended period of elevated influenza mortality risk follows the main waves of influenza pandemics. Soc Sci Med. 2023;328:115975. PubMed https://doi.org/10.1016/j.socscimed.2023.115975

36. Ministry of Health – Manatū Hauora. Clinical rehabilitation guideline for people with long COVID (coronavirus disease) in Aotearoa New Zealand: revised December 2022 [cited 2023

Jan 5]. https://www.health.govt.nz/publication/clinical-rehabilitation-guideline-people-long-covid-coronavirus-disease-aotearoa-new-zealand

37. Wilson N, Boyd M, Nisa S, Clement C, Baker MG. Did exposure to a severe outbreak of pandemic influenza in 1918 impact on long-term survival? Epidemiol Infect. 2016;144:3166–9. PubMed https://doi.org/10.1017/S0950268816001606

38. Patel P, DeCuir J, Abrams J, Campbell AP, Godfred-Cato S, Belay ED. Clinical characteristics of multisystem inflammatory syndrome in adults: a systematic review. JAMA Netw Open. 2021;4:e2126456–2126456. PubMed https://doi.org/10.1001/jamanetworkopen.2021.26456

39. Centers for Disease Control and Prevention. Information for healthcare providers about multisystem inflammatory syndrome in children (MIS-C). 2022 [cited 2022 Dec 29]. https://www.cdc.gov/mis/mis-c/hcp/index.html

40. Kvalsvig A, Brooks A, Wilson N, Bennett J, Summers J, Timu-Parata C, et al. Longer-term harm from Covid-19 in children: The evidence suggests greater efforts are needed to protect children in Aotearoa NZ from infection. 2022 Mar 8 [cited 2022 Dec 29]. https://www.phcc.org.nz/briefing/longer-term-harm-covid-19-children-evidence-suggests-greater-efforts-are-needed-protect

41. Taquet M, Dercon Q, Luciano S, Geddes JR, Husain M, Harrison PJ. Incidence, co-occurrence, and evolution of long-COVID features: a 6-month retrospective cohort study of 273,618 survivors of COVID-19. PLoS Med. 2021;18:e1003773. PubMed https://doi.org/10.1371/journal.pmed.1003773

42. Shereen MA, Khan S, Kazmi A, Bashir N, Siddique R. COVID-19 infection: origin, transmission, and characteristics of human coronaviruses. J Adv Res. 2020;24:91–8. PubMed https://doi.org/10.1016/j.jare.2020.03.005

43. Widdowson M-A, Monto AS. Epidemiology of influenza. In: Webster RG, Monto AS, Braciale TJ, Lamb RA, editors. Textbook of influenza. Oxford: Wiley-Blackwell; 2013. p. 250–66.

44. Greenhalgh T, Jimenez JL, Prather KA, Tufekci Z, Fisman D, Schooley R. Ten scientific reasons in support of airborne transmission of SARS-CoV-2. Lancet. 2021;397:1603–5. PubMed https://doi.org/10.1016/S0140-6736(21)00869-2

45. Jimenez JL, Marr LC, Randall K, Ewing ET, Tufekci Z, Greenhalgh T, et al. What were the historical reasons for the resistance to recognizing airborne transmission during the COVID-19 pandemic? Indoor Air. 2022;32:e13070. PubMed https://doi.org/10.1111/ina.13070

46. Salian VS, Wright JA, Vedell PT, Nair S, Li C, Kandimalla M, et al. COVID-19 transmission, current treatment, and future therapeutic strategies. Mol Pharm. 2021;18:754–71. PubMed https://doi.org/10.1021/acs.molpharmaceut.0c00608

47. Ministry of Business Innovation & Employment - Hikina Whakatutuki, Isolation and quarantine. 2022 [cited 2023 Jan 3]. https://www.mbie.govt.nz/immigration-and-tourism/isolation-and-quarantine

48. Grout L, Katar A, Ait Ouakrim D, Summers JA, Kvalsvig A, Baker MG, et al. Failures of quarantine systems for preventing COVID-19 outbreaks in Australia and New Zealand. Med J Aust. 2021;215:320–4. PubMed https://doi.org/10.5694/mja2.51240

49. Pool DI. The effects of the 1918 pandemic of influenza on the Maori population of New Zealand. Bull Hist Med. 1973;47:273–81. PubMed

50. Summers J, Kvalsvig A, Bennett J, Telfar Barnard L, Baker MG. COVID-19, Christmas, the New Year and Summer Holidays: what the NZ Government and individuals can do to minimise the risks [cited 2023 Jan 5]. https://www.phcc.org.nz/briefing/covid-19-christmas-new-year-and-summer-holidays-what-nz-government-and-individuals-can-do

51. Wilson N, Rice G, Thomson G, Baker M. Re-evaluating a local public health control measure used in New Zealand for the pandemic influenza of 1918. N Z Med J. 2005;118:U1714. PubMed

52. Taubenberger JK, Morens DM. 1918 influenza: the mother of all pandemics. Emerg Infect Dis. 2006;12:15–22. PubMed https://doi.org/10.3201/eid1209.05-0979

53. Bulling GM. Preventable deaths? the 1918 influenza pandemic in Nightcaps [dissertation]. 1991, University of Otago.

54. Ministry of Health, Manatū Hauora. Single case of COVID-19 confirmed in New Zealand. 2020 [cited 2023 Jan 4]. https://www.health.govt.nz/news-media/media-releases/single-case-covid-19-confirmed-new-zealand

55. Lovell Gregg J. How Coromandel kept the influenza epidemic at bay. N Z Med J. 1919;18:51–3.

56. Jamieson JPS. Notes of the recent epidemic of influenza in Nelson. N Z Med J. 1919;18:46–51.

57. New Zealand History–Nga korero a ipurangi o Aotearoa,. Comparing pandemics. 2022 [cited 2023 Jan 4]. https://nzhistory.govt.nz/te-akomanga/contexts-activities/comparing-pandemics

58. Baker M, Summers J, Kvalsvig A, Harwood M, Wilson N. Preparing for Omicron: a proactive Government response is urgently needed to minimise harms. 2022 Jan 16 [cited 2022 Dec 29]. https://www.phcc.org.nz/briefing/preparing-omicron-proactive-government-response-urgently-needed-minimise-harms

59. Ardern J, Verrall A. COVID-19 Protection Framework retired NZ moves forward with certainty. 2022 [cited 2022 Dec 29]. https://www.beehive.govt.nz/release/covid-19-protection-framework-retired-nz-moves-forward-certainty.

60. Ministry of Health – Manatū Hauora,. Summary of Aotearoa New Zealand's Strategic Framework for COVID-19 variants of concern–summary for cabinet. 2022 [cited 2023 Jan 4]. https://www.health.govt.nz/system/files/documents/pages/220601_final_summary_of_voc_sf_for_cabinet_paper_21_june.pdf

61. Tai Tokerau Community Borders. COVID flyer. 2020 [cited 2023 Jan 5]. https://ngapuhi.iwi.nz/wp-content/uploads/2020/03/ZA20-COVID-flyer.pdf

62. Thornber L. Covid-19: Iwi close popular Northland holiday spot over summer. 2021 Nov 29 [cited 2022 Dec 29]. https://www.stuff.co.nz/travel/news/300465501/covid19-iwi-close-popular-northland-holiday-spot-over-summer

63. Te Rūnanga-a-iwi o Ngāti Kahu. 09/12/2021: Maitai campground & Karikari walkway closed for the summers [cited 2023 Jan 2] https://www.ngatikahu.iwi.nz/node/32680

64. National Army Museum of New Zealand–Te Mata Toa. The death ship: a fateful voyage. 2021 [cited 2023 Jan 2]. https://www.armymuseum.co.nz/visit/exhibitions/the-death-ship

65. Ngai Tūhoe. Tu Urewera to remain closed to manuhiri at Alert Level 2 [cited 2023 Jan 4]. https://www.ngaituhoe.iwi.nz/COVID-19-Updates

66. Public Health Agency. COVID-19 mortality in Aotearoa New Zealand: inequities in risk. 2022 Sep. Wellington, New Zealand: Ministry of Health –Manatū Hauora.

67. Ministry of Health–Manatū Hauora. COVID-19 trends and insights report—23 December 2022. 2022 [cited 2023 Jan 10]. https://www.health.govt.nz/system/files/documents/pages/trends_and_insights_report_-_23_december_2022.pdf

68. Wilson N, Barnard LT, Summers JA, Shanks GD, Baker MG. Differential mortality rates by ethnicity in 3 influenza pandemics over a century, New Zealand. Emerg Infect Dis. 2012;18:71–7. PubMed https://doi.org/10.3201/eid1801.110035

69. Steyn N, Binny RN, Hannah K, Hendy SC, James A, Lustig A, et al. Māori and Pacific people in New Zealand have a higher risk of hospitalisation for COVID-19. N Z Med J. 2021;134:28–43. PubMed

70. Wilson N, Baker M. Ninety years on: what we still need to learn from "Black November" 1918 about pandemic influenza. N Z Med J. 2008;121:136–8. PubMed

71. Shanks GD, Wilson N, Kippen R, Brundage JF. The unusually diverse mortality patterns in the Pacific region during the 1918-21 influenza pandemic: reflections at the pandemic's centenary. Lancet Infect Dis. 2018;18:e323–32. PubMed https://doi.org/10.1016/S1473-3099(18)30178-6

72. Rice GW. A disease deadlier than war. N Z Med J. 2013;126:12–4. PubMed

73. Rice G. Maori mortality in the 1918 influenza epidemic. N Z Popul Rev. 1983;9:44–61. PubMed

74. Summers JA, Wilson N, Baker MG. Ethnic gradient in mortality amongst the New Zealand military personnel in World War One. Journal of Epidemiology & Community Health. 2011;65:A466.

75. Rice G. Microgeography of historical influenza mortality: A New Zealand example from the 1918 Pandemic, in 1990, One Thousand Years of New Zealand Population: Heeding the Past and Planning for the Future, in N.Z.D.S Conference. 1989: University of Waikato, Hamilton, New Zealand.

76. Charania NA, Turner NM. 100 years since the 1918 influenza pandemic-progress made, yet questions remain. A synopsis of the 4th New Zealand Influenza Symposium, February 2018. N Z Med J. 2018;131:50–5. PubMed

77. Megget K. How New Zealand's COVID-19 strategy failed the Māori. BMJ. 2022;376:o180. PubMed https://doi.org/10.1136/bmj.o180

78. Sinclair O, Russell J, de Lore D, Andersen E, Percival T, Wiles S. The urgent need for an equitable COVID-19 paediatric vaccine roll-out to protect tamariki Māori. N Z Med J. 2021;134:8–15. PubMed

79. Restrictions on Maoris. Poverty Bay Herald. 1918 Dec 5. p. 6.

80. Public notices: Whangarei Hospital District. Northern Advocate. 1918 Nov 23. p. 3.

81. Menace of Maori gatherings. Press. 1919 Mar 14. p. 6.

82. Influenza epidemic. Hawera & Normanby Star. 1919 Mar 13. p. 7.

83. Hendy, S., N. Steyn, A. James, M.J. Plank, K. Hannah, R.N. Binny, et al., Mathematical modelling to inform New Zealand's COVID-19 response. J R Soc N Z. 2021;51:S86–106.

84. Binny RN, Baker MG, Hendy SC, James A, Lustig A, Plank MJ, et al. Early intervention is the key to success in COVID-19 control. R Soc Open Sci. 2021;8:210488. PubMed https://doi.org/10.1098/rsos.210488

85. Steyn N, Binny RN, Hannah K, Hendy SC, James A, Kukutai T, et al. Estimated inequities in COVID-19 infection fatality rates by ethnicity for Aotearoa New Zealand. N Z Med J. 2020;133:28–39. PubMed https://doi.org/10.1101/2020.04.20.20073437

86. Improvement in camps. Evening Post. 1918 Nov 22. p. 7.

87. Rice GW. Japan and New Zealand in the 1918 influenza pandemic: comparative perspectives on official responses and crisis management. In: Phillips H, Killingray D, editors. The Spanish influenza pandemic of 1918–19. London: Routledge; 2003.

88. Ministry of Health–Manatū Hauora. Treaty of Waitangi principles. 2020 [cited 2023 Jan 4]. https://www.health.govt.nz/our-work/populations/maori-health/he-korowai-oranga/strengthening-he-korowai-oranga/treaty-waitangi-principles

89. King P, Cormack D, McLeod M, Harris R, Gurney J. COVID-19 and Māori health–when equity is more than a word. 2020 Apr 9 [cited 2022 Dec 29]. https://www.phcc.org.nz/briefing/covid-19-and-maori-health-when-equity-more-word

90. McLeod M, Gurney J, Harris R, Cormack D, King P. COVID-19: we must not forget about Indigenous health and equity. Aust N Z J Public Health. 2020;44:253–6. PubMed https://doi.org/10.1111/1753-6405.13015

91. Ministry of Health–Manatū Hauora. COVID-19 Māori Health Protection Plan. 2021. Wellington, New Zealand: Ministry of Health.

92. Cheung G, Bala S, Lyndon M, Ma'u E, Rivera Rodriguez C, Waters DL, et al. Impact of the first wave of COVID-19 on the health and psychosocial well-being of Māori, Pacific Peoples and New Zealand Europeans living in aged residential care. Australas J Ageing. 2021;00:1–8. PubMed

93. Te Rōpū Whakakaupapa Urutā - National Māori Pandemic Group. COVID-19 advice for Māori. 2020 [cited 2023 Jan 3]. https://www.uruta.maori.nz

94. Manuirirangi K, Jarman J. The Taranaki COVID-19 response from a Māori perspective: lessons for mainstream health providers in Aotearoa New Zealand. N Z Med J. 2021;134:122–4. PubMed

95. Rangiwai B, Sciascia AD. The impacts of COVID-19 on tangihanga. Journal of Global Indigeneity. 2021;5:1–14.

96. Rice GW. Why did Wellington suffer nearly double the death rate of Christchurch in the 1918 influenza pandemic? The 2018 Jim Gardner Memorial Lecture. 2018; Canterbury History Foundation: Christchurch, New Zealand.

97. Rice G. Christchurch in the 1918 influenze epidemic: a preliminary study. N Z J Hist. 1979;13:109–37. PubMed

98. Bryder L. “Lessons” of the 1918 influenza epidemic in Auckland. N Z J Hist. 1982;16:97–121. PubMed

99. Silently sanctioned slums. The Oamaru Mail. 1918 Nov 28. p. 3.

100. Wilson N, Mansoor OD, Baker MG. The first analytic evidence for socio-economic gradients in 1918 pandemic influenza mortality rates for New Zealand. N Z Med J. 2018;131:50–3. PubMed

101. McSweeny K, Colman A, Fancourt N, Parnell M, Stantiall S, Rice G, et al. Was rurality protective in the 1918 influenza pandemic in New Zealand? N Z Med J. 2007;120:U2579. PubMed

102. Summers JA, Stanley J, Baker MG, Wilson N. Risk factors for death from pandemic influenza in 1918-1919: a case-control study. Influenza Other Respir Viruses. 2014;8:329–38. PubMed https://doi.org/10.1111/irv.12228

103. Unite against COVID-19. COVID-19 vaccine rollout plan. 2021 [cited 2023 Jan 2]. https://covid19.govt.nz/news-and-data/latest-news/covid-19-vaccine-rollout-plan

104. Bennett J, Anderson P, Donovan S, Kvalsvig A, Leversha A, Rajanaidu S, et al. Strengthening Omicron mitigation strategies in Early Childhood Education settings. 2022 Feb 23 [cited 2023 Jan 5]. https://www.phcc.org.nz/briefing/strengthening-omicron-mitigation-strategies-early-childhood-education-settings

105. Duncanson M, Wheeler BJ, Jelleyman T, Dalziel SR, McIntyre P. Delayed access to care and late presentations in children during the COVID-19 pandemic New Zealand-wide lockdown: A New Zealand Paediatric Surveillance Unit study. J Paediatr Child Health. 2021;57:1600–4. PubMed https://doi.org/10.1111/jpc.15551

106. Jeffs E, Lucas N, Walls T. CoVID-19: Parent and caregiver concerns about reopening New Zealand schools. J Paediatr Child Health. 2021;57:403–8. PubMed https://doi.org/10.1111/jpc.15234

107. Bennett C. “Now the war is over, we have something else to worry us”: New Zealand children’s responses to crises, 1914–1918. JHCY. 2014;7:19–41. https://doi.org/10.1353/hcy.2014.0007

108. Kvalsvig A, Wilson N, Timu-Parata C, Tuari-Toma B, Summers J, Davies C, et al. Protecting New Zealand children from the developing Omicron outbreak. 2022 Jan 25 [cited 2023 Jan 2]. https://www.phcc.org.nz/briefing/protecting-new-zealand-children-developing-omicron-outbreak

109. Russell J, Rajanaidu S, Anderson P, Best E, de Lore D, Jansen RM, et al. Schools and COVID-19 in Aotearoa New Zealand: Keeping schools open as safely as possible. 2022 [cited 2023 Jan 2]. https://cdn.auckland.ac.nz/assets/auckland/news-and-opinion/2022/01/Russell%20et%20al%20%202022%20Schools%20and%20COVID-19%20Keeping%20schools%20open%20(002).pdf

110. Old A. Memo: review of COVID-19 Protection Framework settings–27 July 2022. 2022 [cited 2023 Jan 4]. https://fyi.org.nz/request/20877/response/79906/attach/5/H2022014882%20documents.pdf

111. Kvalsvig A, Wilson N, D'Souza A, Baker M. Children and young people and COVID-19: The urgent need for improved guidance in NZ. 2020 Aug 26 [cited 2022 Dec 29]. https://www.phcc.org.nz/briefing/children-and-young-people-and-covid-19-urgent-need-improved-guidance-nz

112. Crowther S, Maude R, Zhao IY, Bradford B, Gilkison A. New Zealand maternity and midwifery services and the COVID-19 response: a systematic scoping review. Women Birth. 2022;35:213–22. **PMID 34215539**

113. Unite Against COVID-19. If you have COVID-19. 2022 [cited 2023 Jan 2]. https://covid19.govt.nz/testing-and-isolation/if-you-have-covid-19#:~:text=If%20you%20are%20pregnant%20or,a%20mild%20or%20moderate%20symptoms

114. Freeman C, Ergler C, Kearns R, Smith M. COVID-19 in New Zealand and the Pacific: implications for children and families. Child Geogr. 2020.

115. Lange KJ. "Child's play?": the role of children in the 1918 influenza epidemic in New Zealand [thesis]. 2015, Dickinson College.

116. Hatter L, Eathorne A, Hills T, Bruce P, Beasley R. Respiratory syncytial virus: paying the immunity debt with interest. Lancet Child Adolesc Health. 2021;5:e44–5. PubMed https://doi.org/10.1016/S2352-4642(21)00333-3

117. Fitchett F, Bowie JT, Champtaloup ST, Drennan AM. A report on the recent "Influenza Epidemic" in Dunedin. N Z Med J. 1919;18.

118. Franks J. Covid-19: Fears more students will drop out after taking jobs to support whānau. 2021 Sep 2 [cited 2022 Dec 29]. https://www.stuff.co.nz/national/education/126228087/covid19-fears-more-students-will-drop-out-after-taking-jobs-to-support-whnau

119. Wilson N, Turner N, Baker MG. One hundred years ago in 1919: New Zealand's birth reduction shock associated with an influenza pandemic. N Z Med J. 2019;132:57–62. PubMed

120. Chandra S, Christensen J, Mamelund SE, Paneth N. Short-term birth sequelae of the 1918–1920 influenza pandemic in the United States: state-level analysis. Am J Epidemiol. 2018;187:2585–95. PubMed https://doi.org/10.1093/aje/kwy153

121. Mamelund S-E. Can the Spanish influenza pandemic of 1918 explain the baby boom of 1920 in neutral Norway? Population. 2004;59:229–60.

122. Wilson N, Grout L, Summers J, Nghiem N, Baker M. Health and economic impacts of the COVID-19 Response: NZ compared to OECD countries. 2020 Dec 3 [cited 2022 Dec 29]. https://www.phcc.org.nz/briefing/health-and-economic-impacts-covid-19-response-nz-compared-oecd-countries

123. Killick E. Financing the influenza epidemic. Journal of the Department of Public Health, Hospitals, and Charitable Aid. 1919;2:6–12.

124. Robertson G. NZ economy's resilience shown despite COVID impacts. 2021 [cited 2023 Jan 2]. https://www.beehive.govt.nz/release/nz-economy%E2%80%99s-resilience-shown-despite-covid-impacts

125. The Treasury–Te Tai Ōhanga. Fortnightly economic update—18 March 2022. 2022 [cited 2023 Jan]. https://www.treasury.govt.nz/system/files/2022-03/feu-18mar22.pdf

126. Ministry of Health–Manatū Hauora. New Zealand Influenza Pandemic Plan: a framework for action. 2nd ed. Wellington, New Zealand: New Zealand Government–Te Kāwanatanga o Aotearoa: 2017.

127. Baker M, Kvalsvig A, Verrall AJ, Telfar-Barnard L, Wilson N. New Zealand's elimination strategy for the COVID-19 pandemic and what is required to make it work. N Z Med J. 2020;133:10–4. PubMed

128. Baker MG, Kvalsvig A, Verrall AJ. New Zealand's COVID-19 elimination strategy. Med J Aust. 2020;213:198–200.e1. PubMed https://doi.org/10.5694/mja2.50735

129. Baker MG, Kvalsvig A, Crengle S, Harwood M, Tukuitonga C, Betty B, et al. The next phase in Aotearoa New Zealand's COVID-19 response: a tight suppression strategy may be the best option. N Z Med J. 2021;134:8–16. PubMed

130. Jefferies S, French N, Gilkison C, Graham G, Hope V, Marshall J, et al. COVID-19 in New Zealand and the impact of the national response: a descriptive epidemiological study. Lancet Public Health. 2020;5:e612–23. PubMed https://doi.org/10.1016/S2468-2667(20)30225-5

131. Baker MG, Wilson N, Anglemyer A. Successful elimination of Covid-19 transmission in New Zealand. N Engl J Med. 2020;383:e56. PubMed https://doi.org/10.1056/NEJMc2025203

132. Summers J, Cheng H-Y, Lin H-H, Barnard LT, Kvalsvig A, Wilson N, et al. Potential lessons from the Taiwan and New Zealand health responses to the COVID-19 pandemic. Lancet Reg Health West Pac. 2020;4:100044. **PMID 34013216**

133. Baker MG, Wilson N, Blakely T. Elimination could be the optimal response strategy for COVID-19 and other emerging pandemic diseases. BMJ. 2020;371:m4907. PubMed https://doi.org/10.1136/bmj.m4907

134. Kvalsvig A, Baker MG. How Aotearoa New Zealand rapidly revised its Covid-19 response strategy: lessons for the next pandemic plan. J R Soc N Z. 2021;51:S143–66.

135. Baker MG, Durrheim D, Hsu LY, Wilson N. COVID-19 and other pandemics require a coherent response strategy. Lancet. 2023;401:265–6. PubMed https://doi.org/10.1016/S0140-6736(22)02489-8

136. Roddick JA. Delirium and fever in the antipodes: nursing and the 1918 influenza epidemic in Dunedin Hospital. J Res Nurs. 2006;11:357–70. https://doi.org/10.1177/1744987106065832

137. Roddick JA. When the flag flew at half mast: nursing and the 1918 influenza epidemic in Dunedin [thesis]. 2005, Otago Polytechnic.

138. Chan DZL, Stewart RAH, Kerr AJ, Dicker B, Kyle CV, Adamson PD, et al. The impact of a national COVID-19 lockdown on acute coronary syndrome hospitalisations in New Zealand (ANZACS-QI 55). Lancet Reg Health West Pac. 2020;5:100056. PubMed https://doi.org/10.1016/j.lanwpc.2020.100056

139. Gurney JK, Millar E, Dunn A, Pirie R, Mako M, Manderson J, et al. The impact of the COVID-19 pandemic on cancer diagnosis and service access in New Zealand-a country pursuing COVID-19 elimination. Lancet Reg Health West Pac. 2021;10:100127. PubMed https://doi.org/10.1016/j.lanwpc.2021.100127

140. New Zealand Government - Te Kāwanatanga o Aotearoa. Major reforms will make healthcare accessible for all NZers. 2021 Apr 21 [cited 2023 Jan 3]. https://www.beehive.govt.nz/release/major-reforms-will-make-healthcare-accessible-all-nzers

141. Health and Disability System Review. Health and Disability System Review–Final Report–Pūrongo Whakamutunga. Ministry of Health. 2020. Wellington, New Zealand.

142. Wood PJ. Managing boundaries between professional and lay nursing following the influenza pandemic, 1918–1919: insights for professional resilience today? J Clin Nurs. 2017;26:805–12. PubMed https://doi.org/10.1111/jocn.13570

143. Citizens Advice Bureau. What are my rights as an employee, with Covid-19 vaccination? 2022 [cited 2023 Jan 3]. https://www.cab.org.nz/article/KB00041126

144. 1920 New Zealand Health Act. New Zealand Government—Te Kāwanatanga o Aotearoa: Wellington, New Zealand.

145. Epidemic commission. New Zealand Herald. 1919 Feb 22. p. 10.

146. Rice GW. The making of New Zealand's 1920 Health Act. N Z J Hist. 1988;22:3–22. PubMed

147. New Zealand Government - Te Kāwanatanga o Aotearoa - Post-Cabinet Press Conference: Monday, 5 December 2022—Hansard Transcript. 2022 Dec 5 [cited 2023 Jan 4]. https://www.beehive.govt.nz/sites/default/files/2022-12/Press%20Conference%205%20December%202022.pdf

148. New Zealand Government - Te Kāwanatanga o Aotearoa . Summary of the terms of reference for the Royal Commission of Inquiry into lessons learned from Aotearoa New Zealand's response to COVID-19 that should be applied in preparation for a future pandemic [cited 2023 Jan 3]. https://www.beehive.govt.nz/sites/default/files/2022-12/Summary%20of%20ToR%20for%20Royal%20Commission%20into%20COVID%20and%20any%20future%20pandemic.pdf

149. Eyre JWH, Lowe EC. Report upon the Autumn Influenza Epidemic (1918) as it affected the N.Z.E.F. in the United Kingdom. Lancet. 1919;1919:553–60. https://doi.org/10.1016/S0140-6736(01)25676-1

150. Eyre JWH, Lowe CE. Prophylactic vaccinations. Lancet. 1918;12:484–7. https://doi.org/10.1016/S0140-6736(01)02840-9

151. Armitage FL. Note on "Influenza" and pneumonia: from a field hospital. BMJ. 1919;1:272–4. PubMed https://doi.org/10.1136/bmj.1.3036.272

152. Chien YW, Klugman KP, Morens DM. Efficacy of whole-cell killed bacterial vaccines in preventing pneumonia and death during the 1918 influenza pandemic. J Infect Dis. 2010;202:1639–48. PubMed https://doi.org/10.1086/657144

153. Summers J. An Overview of Vaccine use amongst the New Zealand Expeditionary Forces during World War One [dissertation]. 2020, The Worshipful Society of Apothecaries.

154. Efficiency of influenza inoculation. Nelson Evening Mail. 1918 Dec 31. p. 6.

155. Immunisation Advisory Centre. Vaccine development. 2022 [cited 2023 Jan 2]. https://www.immune.org.nz/vaccines/vaccine-development/brief-history-vaccination

156. Thaker J. The persistence of vaccine hesitancy: COVID-19 vaccination intention in New Zealand. J Health Commun. 2021;26:104–11. PubMed https://doi.org/10.1080/10810730.2021.1899346

157. Radio New Zealand. Covid-19: High Court quashes ‘unlawful’ vaccine mandate for police and defence force staff. 2022 [cited 2023 Jan 4]. https://www.rnz.co.nz/news/national/462265/covid-19-high-court-quashes-unlawful-vaccine-mandate-for-police-and-defence-force-staff

158. New Zealand Herald. Covid 19 Omicron: defence, police vaccine mandates—Government appeals High Court decision. 2022 Mar 25 [cited 2023 Jan 4]. https://www.nzherald.co.nz/nz/covid-19-omicron-defence-police-vaccine-mandates-government-appeals-high-court-decision/RM44FQY5HEW26QX3IVFJYHKD7U/.

159. New Zealand Foreign Affairs and Trade - Manatū Aorere. Apia: Our Story [cited 2023 Jan 2]. https://www.mfat.govt.nz/en/about-us/mfat75/75-our-story/apia

160. Wilson N, Rice G, Thomson G, Baker MG. Remembering past pandemics to ensure we plan for the future: updated New Zealand survey of memorials to the 1918 influenza pandemic. N Z Med J. 2021;134:111–4. PubMed
